# Impact of type 2 diabetes on the relationship between chronic kidney disease and cardiovascular outcomes in heart failure across ejection fraction: observational study from the Swedish heart failure and the Swedish National diabetes registries

**DOI:** 10.1186/s12933-025-02998-w

**Published:** 2025-12-01

**Authors:** Aurora Merolla, Valeria Valente, Christian Basile, Lina Benson, Francesco Cosentino, Ulf Dahlström, Soffia Gudbjörnsdottir, Patrizia Rovere-Querini, Lars H. Lund, Gianluigi Savarese, Giulia Ferrannini

**Affiliations:** 1 Division of Cardiology, Department of Medicine, Karolinska Institutet, Eugeniavägen 27, 111 11 Stockholm, Solna, Sweden; 2 Vita-Salute San Raffaele University, Milan, Italy; 3 Department of Clinical Science and Education, Karolinska Institute, Södersjukhuset, Stockholm, Sweden; 4 Heart and Vascular Theme, Karolinska University Hospital Solna, Stockholm, Sweden; 5 Department of Cardiology, Department of Health, Medicine and Caring Sciences, Linköping University Hospital, Linköping University, Linköping, Sweden; 6 National Diabetes Registry, Centre of Registries, Gothenburg, Sweden; 7 Department of Molecular and Clinical Medicine, Institute of Medicine, University of Gothenburg, Gothenburg, Sweden; 8 IRCCS San Raffaele Hospital, Milan, Italy; 9 Internal Medicine Unit, Södertälje hospital, Södertälje, Sweden

**Keywords:** Heart failure, Chronic kidney disease, Type 2 diabetes, Ejection fraction, Registry-based research

## Abstract

**Background:**

Chronic kidney disease (CKD) is a risk factor for cardiovascular (CV) events in patients with heart failure (HF). It is unclear whether type 2 diabetes (T2D), closely intertwined with both HF and CKD, modifies the association between cardiovascular outcomes and CKD in HF patients, and whether this association differs according to ejection fraction (EF).

**Methods:**

HF patients enrolled in the Swedish Heart Failure Registry from January 2017 to December 2021 were analyzed. Linkage with the National Diabetes Registry and other population registries provided extensive baseline information. Patients were stratified by T2D status and CKD stages, defined by estimated glomerular filtration rate (eGFR: <30, 30–44, 45–59, ≥ 60 ml/min/1.73 m^2^). The primary outcome was the composite of time to first HF hospitalization (HHF) or CV death. Secondary outcomes were major adverse CV events (MACE, i.e. CV death, non-fatal myocardial infarction and stroke), CV death and all-cause death. Multivariable Cox regression models assessed the associations between eGFR and outcomes according to T2D, including interaction testing. A subgroup analysis was conducted by EF.

**Results:**

Of 36,597 patients included, 8,053 (22%) had T2D, 23,562 (64.4%), 7122 (19.4%), 4477 (12.2%), 1436 (4.0%), were in the four eGFR categories (eGFR ≥ 60, 45–59, 30–44, and < 30 mL/min/1.73 m^2^, respectively), and 53%, 25%, 22% had HF with reduced, mildly-reduced, and preserved EF, respectively. Across eGFR, patients with vs. without T2D were younger, more often male, with higher CV comorbidity and more frequent use of cardio-renal drugs. Across EF categories, T2D patients had higher prevalence of CKD. Lower eGFR categories were progressively associated with higher risk of the primary outcome, independently of T2D. This was consistent across EF, except in HFpEF with eGFR < 30 ml/min/1.73 m^2^, where the magnitude of the association in T2D group was smaller than in non-T2D (*p*-interaction < 0.01). Risks of MACE, CV death and all-cause mortality were higher for lower eGFR categories, with lower hazards in T2D group (*p*-interaction < 0.01).

**Conclusions:**

In a contemporary HF cohort, decreased kidney function was associated with a progressively higher risk of HHF/CV death, and T2D was not a risk modifier. Renal protection should therefore be implemented in HF regardless of T2D.

**Supplementary Information:**

The online version contains supplementary material available at 10.1186/s12933-025-02998-w.

## Research insights

What is currently known about this topic?

Heart failure (HF), kidney disease and diabetes often coexist and worsen each other’s prognosis.

Real-world evidence on large contemporary cohorts addressing this complex interplay and evaluating clinical outcomes is lacking.

What is the key research question?

Is type 2 diabetes (T2DM) a risk modifier of the association between chronic kidney disease and outcomes in HF?

What is new?

This study includes a large HF population, with all different classes represented and with a large number of clinical characteristics.

T2DM was not a risk modifier of the association between HF-related outcomes and CKD in patients with HF.

We examine many subgroups.

How might this study influence clinical practice?

Multicomorbid patients with HF, CKD and T2DM deserve a tailored approach and careful evaluation of renal function to appropriately plan related therapies.

## Background

Heart failure (HF) is a major public health concern associated with high resource use and healthcare costs [1]. Affecting over 64 million people worldwide, HF is the leading cause of hospitalization in patients over 65 years of age, with numbers increasing over time and 1-year mortality risk ranging 15–30% [1].

HF often coexists with type 2 diabetes mellitus (T2D) and chronic kidney disease (CKD), with approximately 40–50% of HF patients having CKD, 25–40% having T2D and 16% having HF, CKD and T2D [2]. Despite being heterogeneous diseases, HF, CKD and T2D share a common substrate of risk factors and pathophysiological pathways, including insulin resistance, inflammation and neuro-hormonal activation, with each comorbidity contributing to the onset and worsening the outcome of the others in a vicious circle [3, 4]. CKD is related to poor prognosis in HF across all ranges of EF, with even higher rates of HF hospitalizations (HHF) for lower levels of estimated glomerular filtration rate (eGFR) [5, 6].

T2D is well known to be associated with incident HF and with cardiovascular (CV) morbidity and mortality in prevalent HF, regardless of ejection fraction (EF) [7]. Real-world data underscore higher CV-related hospitalization rates in patients with comorbid HF, T2D, and CKD compared to those with HF alone, which may result in high mortality and significant healthcare costs [8].

Real-world evidence on large contemporary cohorts addressing this complex interplay and evaluating clinical outcomes is lacking. The recent introduction of treatment options with proven benefits on cardiorenal outcomes in HF regardless of the presence of diabetes [(i.e. sacubitril/valsartan, sodium-glucose cotransporter-2 inhibitors (SGLT2i), and non-steroidal mineralocorticoid receptor antagonists (MRA)] calls for its better characterization [9–11].

Here, we aim to investigate the association of CKD with HF and CV outcomes according to CKD categories and to assess whether T2D may be a risk modifier in CKD patients.

## Methods

### Data sources

We analyzed data from the Swedish Heart Failure Registry (SwedeHF), a national quality registry established in 2000 with ongoing enrolment of HF patients from secondary care in- and out-patient wards or clinics and, to a limited extent, primary care centers. SwedeHF has been further described previously [12]. The inclusion criterion was based on clinician judgment until April 2017. Thereafter, HF diagnosis was assigned according to ICD-10 codes I50.0, I50.1, I50.9, I42.0, I42.6, I42.7, I25.5, I11.0, I13.0 and I13.2. We included patients from 1 January 2017 to 31 December 2021. Information on T2D were obtained by linking the SwedeHF to the Swedish National Diabetes Registry (NDR, www.ndr.nu), a nationwide registry including patients with diabetes from primary care visits and outpatient clinics nationwide [13]. Additional data on baseline comorbidities and outcomes were obtained from the National Patient Register (NPR) according to International Classification of Diseases (ICD-10). SwedeHF was also linked to the Cause of Death Register, for retrieving information on date and cause of death, to the National Prescribed Drug Register, which contains data for all dispensed prescriptions since 2005, and to the longitudinal integrated database for health insurance and labour market studies (LISA), a register providing socioeconomic characteristics as income, level of education and living environment. The unique personal identification number, held by all Swedish residents, enables the linkage across the above-mentioned national administrative and quality registries. This analysis and the linkage across the above-mentioned data sources was approved by the Swedish Ethical Review Authority and complies with the Declaration of Helsinki. In SwedeHF, written informed consent is not required, but patients are informed of their registration and allowed to opt-out. For NDR, each patient provides oral or written informed consent for inclusion in the registry.

### Definitions

For our analysis the index date was defined as the registration date in SwedeHF registry, date of visit for out-patients and date of discharge for in-patients. In case of multiple registrations, the first one was considered.

In SwedeHF, the definition of HF was based on clinician judgment until April 2017. Thereafter, HF diagnosis was assigned according to ICD-10 codes I50.0, I50.1, I50.9, I42.0, I42.6, I42.7, I25.5, I11.0, I13.0 and I13.2.

T2D was defined as fulfilling any of the below:

- T2D registered in NDR prior to index registration in SwedeHF. Since only year of onset, and not exact date, is recorded in the NDR, we made the assumption, that if the patient's index date fell in the first half of the year (1 January–30 June), the year of onset of T2D needed to be in the previous year to be considered as prior T2D. Otherwise, i.e. if it fell in the second half (1 July–31 December), the onset year could be the same year as the index date to be considered as prior;
- recorded as having T2D in SwedeHF;
- T2D as comorbidity prior to index date in NPR.

A control population with HF but without T2D was selected from SwedeHF as not fulfilling the above-mentioned criteria for T2D diagnosis. Patients with type 1 diabetes or other/missing types of diabetes, those with missing data for EF and eGFR, and those who died during the index hospitalization were excluded.

CKD categories were defined according to eGFR and based on the Kidney Disease Improving Global Outcomes classification: normal or mildly decreased (eGFR ≥ 60 ml/min/1.73 m^2^), mildly to moderately (eGFR 45–59 ml/min/1.73m^2^), moderately to severely (eGFR 30–44 ml/min/1.73m^2^), and severely decreased (eGFR < 30 ml/min/1.73 m^2^) [14]. EGFR was estimated using the 2021 CKD Epidemiology Collaboration (CKD-EPI) equation based on serum or plasma creatinine collected at the index date.

All variables definitions and ICD-10 codes used to define comorbidities and outcomes are reported in the supplementary Table *S1*.

A detailed flowchart of the population selection is illustrated in the supplementary Figure S1.

### Statistical analysis

The population was stratified according to T2D status and eGFR categories as specified above. Baseline characteristics were summarized by medians (interquartile range-IQR) for continuous variables and by frequency (percentage) for categorical variables, then compared by the Mann–Whitney test and the chi-square test, respectively.

The primary outcome was the composite of time to first HHF or CV death. Secondary outcomes were time to first major adverse CV events (MACE), which included a composite of CV death, non-fatal myocardial infarction (MI) and non-fatal stroke/transient ischemic attack (TIA), single MACE components, all-cause death and repeated HHF. Data were censored at the end of follow-up, emigration from Sweden or death.

Incidence rates (IR) in both T2D and non-T2D groups and IR ratios (IRR) were calculated for each outcome, and the Mantel–Haenszel (M-H) test was applied to test for heterogeneity across eGFR ranges.

Multiple imputation (10 imputed datasets generated) was used to handle missing values in variables which were required for multivariable models. Variables included in multiple imputation model are reported in Table 1 (labelled with the latter *a*), whereas online supplementary Table S2 shows the percentage of missing records per baseline variable.

Multivariable Cox proportional hazards regression models were performed to assess the independent associations between eGFR strata, compared to the reference category ≥ 60 ml/ min/1.73 m^2^, and outcomes, according to T2D and adjusting for covariates specified in Table 1 with the letter *b*. Proportional hazards assumptions were tested for each model with Schoenfelds residuals and visual assessment. Tests for interactions between T2D and eGFR were included in the models and carried out for each outcome. The association with repeated HHF was assessed by negative binomial regression, after verifying the overdispersion of the data.

**Table 1:** Baseline characteristics of HF population with and without T2D across eGFR ranges

	Overall				eGFR < 30 ml/min/1.73 m2			eGFR 30–44 ml/min/1.73 m2			eGFR 45–59 ml/min/1.73 m2			eGFR ≥ 60 ml/min/1.73 m2		
	Overall	no T2D	T2D	*p*-value	no T2D	T2D	*p*-value	no T2D	T2D	*p*-value	no T2D	T2D	*p*-value	no T2D	T2D	*p*-value
Patients, n (%)	36,597 (100)	28,544 (78.0)	8053 (22.0)		959 (66.8)	477 (33.2)		3093 (69.0)	1384 (31.0)		5410 (76.0)	1712 (24.0)		19,082 (81.0)	4480 (19.0)	
*Demographics and socioeconomics*																
Age (years), median (IQR)^b^	75 (66,81)	75 (65,82)	75 (68,80)	0.084	82 (76,87)	78 (72,83)	< 0.001	82 (76,86)	78 (73,83)	< 0.001	79 (73,84)	77 (72,82)	< 0.001	71 (61,78)	72 (65,78)	< 0.001
Female sex, n (%)^b^	12,603 (34.4)	10,245 (35.9)	2358 (29.3)	< 0.001	453 (47.2)	196 (41.1)	0.028	1425 (46.1)	521 (37.6)	< 0.001	2251 (41.6)	549 (32.1)	< 0.001	6116 (32.1)	1092 (24.4)	< 0.001
Family type, n (%)^a,b^				0.60			0.12			0.006			0.13			0.059
Cohabitating	19,654 (53.8)	15,350 (53.8)	4304 (53.5)		435 (45.4)	237 (49.7)		1451 (46.9)	711 (51.4)		2812 (52.0)	925 (54.1)		10,652 (55.9)	2431 (54.3)	
Living alone	16,905 (46.2)	13,164 (46.2)	3741 (46.5)		524 (54.6)	240 (50.3)		1640 (53.1)	673 (48.6)		2594 (48.0)	785 (45.9)		8406 (44.1)	2043 (45.7)	
Education level, n (%)^a,b^				< 0.001			0.42			0.036			< 0.001			< 0.001
Compulsory school	12,768 (35.3)	9558 (33.9)	3210 (40.5)		411 (43.3)	212 (44.8)		1366 (44.8)	612 (45.1)		2065 (38.6)	728 (43.4)		5716 (30.3)	1658 (37.6)	
Secondary school	15,739 (43.6)	12,298 (43.6)	3441 (43.5)		367 (38.7)	189 (40.0)		1149 (37.7)	546 (40.3)		2185 (40.8)	695 (41.4)		8597 (45.6)	2011 (45.6)	
University	7618 (21.1)	6352 (22.5)	1266 (16.0)		171 (18.0)	72 (15.2)		536 (17.6)	198 (14.6)		1101 (20.6)	256 (15.2)		4544 (24.1)	740 (16.8)	
Income ≥ median, n (%)^ab^	18,294 (50.0)	14,779 (51.8)	3515 (43.7)	< 0.001	344 (35.9)	175 (36.7)	0.76	1248 (40.4)	505 (36.5)	0.014	2411 (44.6)	690 (40.4)	0.002	10,776 (56.5)	2145 (47.9)	< 0.001
*Clinical variables*																
EF, n (%)^b^				< 0.001			0.016			0.93			0.38			< 0.001
HFrEF	19,175 (52.4)	14,975 (52.5)	4200 (52.2)		499 (52.0)	210 (44.0)		1443 (46.7)	638 (46.1)		2663 (49.2)	871 (50.9)		10,370 (54.3)	2481 (55.4)	
HFmrEF	9304 (25.4)	7388 (25.9)	1916 (23.8)		204 (21.3)	121 (25.4)		749 (24.2)	336 (24.3)		1375 (25.4)	409 (23.9)		5060 (26.5)	1050 (23.4)	
HFpEF	8118 (22.2)	6181 (21.7)	1937 (24.1)		256 (26.7)	146 (30.6)		901 (29.1)	410 (29.6)		1372 (25.4)	432 (25.2)		3652 (19.1)	949 (21.2)	
NYHA class, n (%)^a,b^				< 0.001			0.81			0.097			< 0.001			< 0.001
I	3373 (11.9)	2881 (13.0)	492 (8.0)		24(3.8)	16(5.1)		130 (5.8)	59(5.8)		352 (8.4)	77(5.8)		2375 (15.8)	340 (9.7)	
II	14,439 (51.1)	11,575 (52.4)	2864 (46.5)		211 (33.7)	109 (34.5)		961 (42.5)	390 (38.2)		2064 (49.4)	552 (41.9)		8339 (55.5)	1813 (51.8)	
III	10,064 (35.6)	7383 (33.4)	2681 (43.6)		369 (58.9)	180 (57.0)		1121 (49.6)	543 (53.2)		1701 (40.8)	659 (50.0)		4192 (27.9)	1299 (37.1)	
IV	369 (1.3)	252 (1.1)	117 (1.9)		23(3.7)	11(3.5)		47(2.1)	28(2.7)		57(1.4)	30(2.3)		125 (0.8)	48(1.4)	
NT-proBNP (pg/ml), median (IQR) ^a,b^	1860 (767, 4060)	1870 (750, 4065)	1817 (826, 4050)	0.25	7161 (3070, 18,400)	5581 (2412, 13,400)	< 0.001	3540 (1760, 7395)	2852 (1292, 5986)	< 0.001	2480 (1180, 4978)	2160 (1045, 4460)	< 0.001	1430 (559, 3060)	1357 (608, 2840)	0.45
BMI (kg/m^2^), median (IQR) ^a,b^	26.8 (23.7, 30.5)	26.2 (23.3, 29.7)	29.0 (25.6, 33.0)	< 0.001	25.2 (22.5, 28.9)	29.3 (25.8, 32.6)	< 0.001	25.6 (22.9, 29.1)	29.6 (25.8, 33.7)	< 0.001	26.0 (23.1, 29.4)	28.9 (25.6, 32.5)	< 0.001	26.4 (23.5, 30.1)	28.8 (25.6, 33.0)	< 0.001
*Medical History*																
Smoking, n(%)^a,b^				< 0.001			0.32			0.030			< 0.001			< 0.001
Never	11,711 (43.6)	9469 (45.0)	2242 (38.5)		316 (50.9)	146 (47.4)		1057 (48.4)	424 (44.2)		1872 (47.0)	492 (39.5)		6224 (43.6)	1180 (35.7)	
Current/Former	15,172 (56.4)	11,596 (55.0)	3576 (61.5)		305 (49.1)	162 (52.6)		1129 (51.6)	536 (55.8)		2110 (53.0)	754 (60.5)		8052 (56.4)	2124 (64.3)	
Alcohol consumption, n (%)^b^	1204 (3.3)	986 (3.5)	218 (2.7)	< 0.001	18(1.9)	6(1.3)	0.39	44(1.4)	22(1.6)	0.67	101 (1.9)	30(1.8)	0.76	823 (4.3)	160 (3.6)	0.025
Duration of HF, n (%)^a,b^				< 0.001			0.75			0.057			0.003			< 0.001
< 6 months	17,397 (49.1)	14,105 (51.0)	3292 (42.4)		331 (35.9)	161 (35.0)		1062 (35.5)	434 (32.5)		2149 (41.0)	608 (36.9)		10,563 (57.2)	2089 (48.4)	
> 6 months	17,999 (50.9)	13,533 (49.0)	4466 (57.6)		592 (64.1)	299 (65.0)		1933 (64.5)	902 (67.5)		3092 (59.0)	1038 (63.1)		7916 (42.8)	2227 (51.6)	
Previous HHF within 1 year, n(%)^b^	15,531 (42.4)	11,712 (41.0)	3819 (47.4)	< 0.001	608 (63.4)	340 (71.3)	0.003	1611 (52.1)	794 (57.4)	0.001	2288 (42.3)	824 (48.1)	< 0.001	7205 (37.8)	1861 (41.5)	< 0.001
Ischemic heart disease, n(%)^b^	17,547 (47.9)	12,568 (44.0)	4979 (61.8)	< 0.001	492 (51.3)	306 (64.2)	< 0.001	1655 (53.5)	922 (66.6)	< 0.001	2670 (49.4)	1137 (66.4)	< 0.001	7751 (40.6)	2614 (58.3)	< 0.001
Atrial Fibrillation, n (%)^b^	20,717 (56.6)	16,089 (56.4)	4628 (57.5)	0.078	605 (63.1)	280 (58.7)	0.11	2140 (69.2)	868 (62.7)	< 0.001	3564 (65.9)	1079 (63.0)	0.031	9780 (51.3)	2401 (53.6)	0.005
Arterial Hypertension, n (%)^b^	25,189 (68.8)	18,223 (63.8)	6966 (86.5)	< 0.001	814 (84.9)	452 (94.8)	< 0.001	2457 (79.4)	1262 (91.2)	< 0.001	3938 (72.8)	1520 (88.8)	< 0.001	11,014 (57.7)	3732 (83.3)	< 0.001
Renal Failure, n (%)^b^	5112 (14.0)	3274 (11.5)	1838 (22.8)	< 0.001	703 (73.3)	399 (83.6)	< 0.001	1110 (35.9)	705 (50.9)	< 0.001	770 (14.2)	448 (26.2)	< 0.001	691 (3.6)	286 (6.4)	< 0.001
Peripheral artery disease, n (%)^b^	3074 (8.4)	2167 (7.6)	907 (11.3)	< 0.001	143 (14.9)	88 (18.4)	0.086	325 (10.5)	174 (12.6)	0.042	472 (8.7)	207 (12.1)	< 0.001	1227 (6.4)	438 (9.8)	< 0.001
Cerebrovascular disease, n (%)^b^	5282 (14.4)	3863 (13.5)	1419 (17.6)	< 0.001	194 (20.2)	105 (22.0)	0.43	559 (18.1)	276 (19.9)	0.14	890 (16.5)	322 (18.8)	0.024	2220 (11.6)	716 (16.0)	< 0.001
COPD, n (%)^b^	4340 (11.9)	3249 (11.4)	1091 (13.5)	< 0.001	143 (14.9)	58 (12.2)	0.16	443 (14.3)	238 (17.2)	0.013	727 (13.4)	243 (14.2)	0.43	1936 (10.1)	552 (12.3)	< 0.001
*Concomitant medications*																
Beta-blockers, n (%)^b^	33,044 (90.3)	25,639 (89.8)	7405 (92.0)	< 0.001	866 (90.3)	433 (90.8)	0.77	2794 (90.3)	1260 (91.0)	0.45	4900 (90.6)	1579 (92.2)	0.037	17,079 (89.5)	4133 (92.3)	< 0.001
RASi or ARNI, n (%)^b^	32,875 (89.8)	25,595 (89.7)	7280 (90.4)	0.055	708 (73.8)	346 (72.5)	0.60	2594 (83.9)	1191 (86.1)	0.061	4803 (88.8)	1561 (91.2)	0.005	17,490 (91.7)	4182 (93.3)	< 0.001
MRA, n (%)^b^	17,751 (48.5)	13,616 (47.7)	4135 (51.3)	< 0.001	295 (30.8)	142 (29.8)	0.70	1447 (46.8)	604 (43.6)	0.051	2790 (51.6)	915 (53.4)	0.18	9084 (47.6)	2474 (55.2)	< 0.001
Diuretics, n (%)^b^	24,701 (67.5)	18,410 (64.5)	6291 (78.1)	< 0.001	865 (90.2)	444 (93.1)	0.070	2666 (86.2)	1253 (90.5)	< 0.001	4062 (75.1)	1439 (84.1)	< 0.001	10,817 (56.7)	3155 (70.4)	< 0.001
Antiplatelets, n (%)^b^	13,181 (36.0)	9569 (33.5)	3612 (44.9)	< 0.001	340 (35.5)	220 (46.1)	< 0.001	987 (31.9)	606 (43.8)	< 0.001	1724 (31.9)	706 (41.2)	< 0.001	6518 (34.2)	2080 (46.4)	< 0.001
Anticoagulants, n (%)^b^	20,578 (56.2)	16,054 (56.2)	4524 (56.2)	0.92	532 (55.5)	251 (52.6)	0.31	2007 (64.9)	822 (59.4)	< 0.001	3448 (63.7)	1046 (61.1)	0.049	10,067 (52.8)	2405 (53.7)	0.26
Lipid-lowering therapy, n (%)^b^	19,537 (53.4)	13,378 (46.9)	6159 (76.5)	< 0.001	464 (48.4)	361 (75.7)	< 0.001	1512 (48.9)	1040 (75.1)	< 0.001	2647 (48.9)	1332 (77.8)	< 0.001	8755 (45.9)	3426 (76.5)	< 0.001
Insulin, n (%)^b^	3218 (8.8)	0(0.0)	3218 (40.0)	< 0.001	0(0.0)	291 (61.0)	< 0.001	0(0.0)	755 (54.6)	< 0.001	0(0.0)	712 (41.6)	< 0.001	0(0.0)	1460 (32.6)	< 0.001
SGLT2i, n (%)^b^	1794 (4.9)	584 (2.0)	1210 (15.0)	< 0.001	3(0.3)	12(2.5)	< 0.001	51(1.6)	115 (8.3)	< 0.001	89(1.6)	229 (13.4)	< 0.001	441 (2.3)	854 (19.1)	< 0.001
GLP1-RA, n (%)^b^	714 (2.0)	9(< 1)	705 (8.8)	< 0.001	2(0.2)	25(5.2)	< 0.001	0(0.0)	119 (8.6)	< 0.001	3(0.1)	158 (9.2)	< 0.001	4(< 1)	403 (9.0)	< 0.001

eGFR, estimated glomerular filtration rate HFrEF, heart failure with reduced ejection fraction; HFmrEF, heart failure with mildly reduced ejection fraction; HFpEF, heart failure with preserved ejection fraction; NYHA, New York Heart Association; NT-proBNP, N-terminal pro B-type natriuretic peptide; MAP, mean arterial pressure; HHF, hospitalization for heart failure; bpm, beats per minute; BMI, body mass index; COPD, chronic obstructive pulmonary disease; RASi, renin-angiotensin antagonists system; ARNI, angiotensin receptor-neprilysin inhibitors; MRA, mineralocorticoid receptor antagonists; SGLT2i, sodium-glucose cotransporter 2 inhibitors; GLP-1 RA, glucagon-like peptide 1 receptor agonists

^a^Variables used for multiple imputation

^b^Variables included in the multiadjusted Cox regression model

Furthermore, a sub-analysis was conducted by EF categories, classified as HF with reduced EF (HFrEF, EF < 40%), mildly reduced EF (HFmrEF, EF 40–49%), and preserved EF (HFpEF, EF ≥ 50%).

Statistical analyses were performed by Stata18.0 (StataCorp. 2023. Stata Statistical Software: Release 18. College Station, TX: StataCorp LLC.). A two-sided *p*-value < 0.05 was considered statistically significant.

## Results

### Baseline characteristics

Baseline characteristics of the study population are shown in Table 1 and in Supplementary Table S3. A total of 36,597 HF patients, of which 8,053 (22.0%) with T2D, were included in the analyses. The population comprised 19,175 patients (52.4%) with HFrEF, 9,304 (25.4%) and 8,118 (22.2%) with HFmrEF and HFpEF, respectively. T2D patients had a higher prevalence of HFpEF compared to non-diabetics (24.1% vs 21.7%, respectively, *p* < 0.001). Overall, the eGFR distribution was as follows: 64.4% with eGFR of 60 ml/min/1.73 m^2^ or higher, 19.4% had eGFR between 45–59 ml/min/1.73m^2^, 12.2% between 30–44 ml/min/1.73 m^2^ and 4.0% with eGFR less than 30 ml/min/1.73 m^2^. The prevalence of T2D was progressively higher as eGFR decreased (from 19.0% in eGFR range ≥ 60 ml/min/1.73m^2^ to 33.2% in eGFR < 30 ml/min/1.73m^2^, as shown in Table 1 and Supplementary Figure S2). In each eGFR category lower than 60 ml/min/1.73m^2^, the proportion of T2D patients was higher compared to non-T2D, in the overall population and across EF subgroups (Supplementary Tables S4 – S6).

The overall median age was 75 (66;81) years. Across eGFR, T2D patients were younger than non-T2D, except for those with eGFR ≥ 60 ml/min/1.73m^2^. They were also more likely to be male and current/former smokers. There was a higher prevalence of comorbidities in T2D-group, including obesity, arterial hypertension, ischemic heart disease, previous acute kidney failure or HHF within 1 year, and longer duration of HF. Significant differences between the two groups were also observed in concomitant medications, with higher use of beta-blockers, renin-angiotensin system inhibitors (RAASi) and angiotensin receptor-neprilysin inhibitors (ARNi) in T2D patients with eGFR 45–59 and ≥ 60 ml/min/1.73m^2^, mineralocorticoid receptor antagonists in T2D with eGFR ≥ 60 ml/min/1.73m^2^ and SGLT2i in T2D across all eGFR categories.

### Outcomes

#### Primary outcome

In the overall HF population, over a median follow-up of 1.8 (0.64–3.1) years, the crude incidence rate (IR) in T2D-group vs non-T2D was 19.4 vs 12.8 per 100 person-years, with an IRR of 1.51 (95%CI 1.45–1.58, *p* < 0.001). The M-H test showed significant heterogeneity across eGFR ranges, with a combined IRR of 1.35 (95% CI 1.29–1.41, *p* < 0.001).

**Fig. 1:**
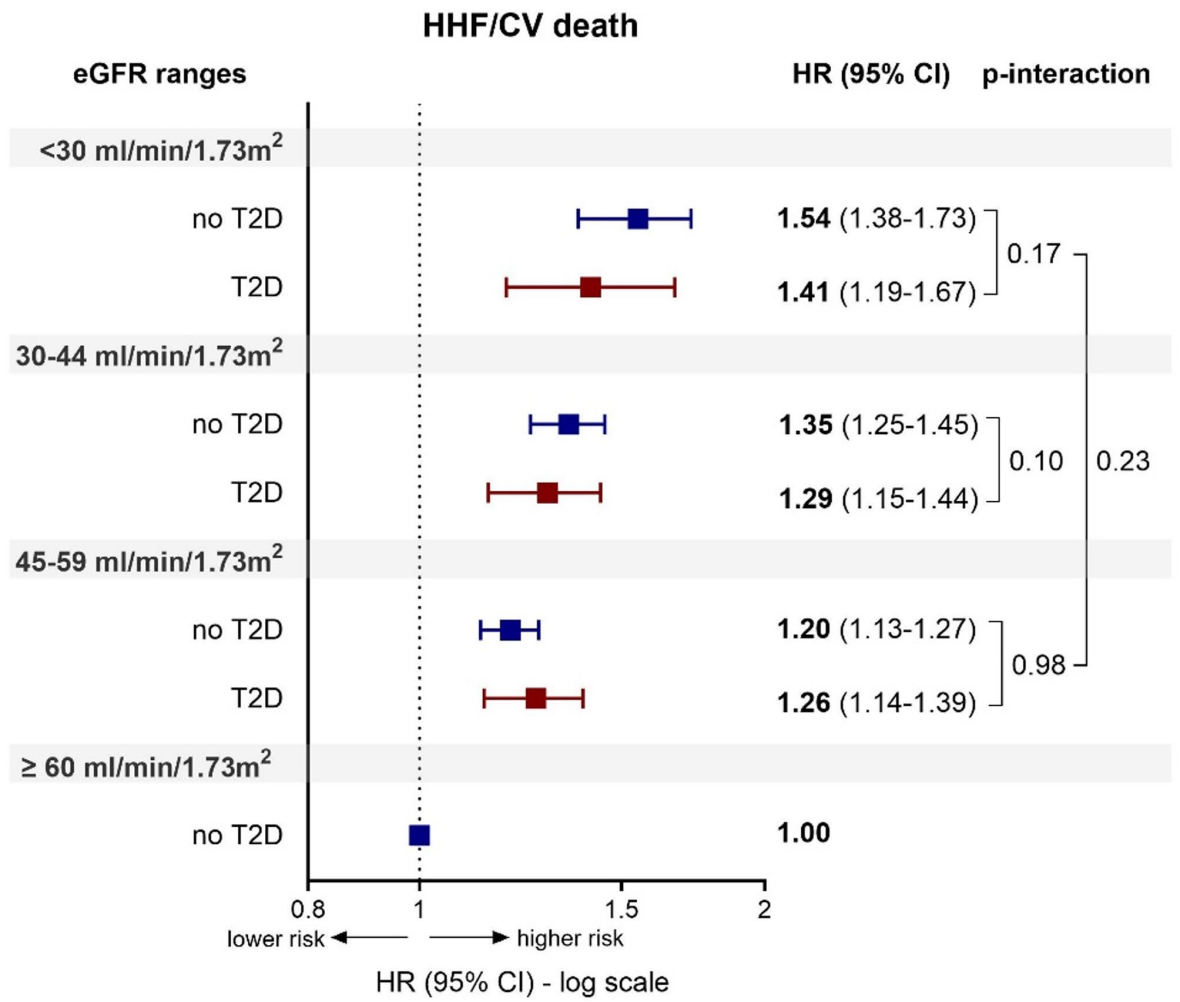
Association of eGFR ranges with the composite primary outcome of HHF and CV death in the overall population according to T2D. HR of each eGFR range (< 30, 30–44, 45–59 ml/min/1.73 m^2^) is relative to the eGFR reference category ≥ 60 ml/min/1.73m^2^ of non T2D group (HR = 1). HHF, hospitalization for heart failure; CV, cardiovascular; eGFR, estimated glomerular filtration rate; T2D, Type 2 Diabetes; HR, Hazard Ratio; CI, confidence interval

In multivariable Cox regression analysis, lower eGFR ranges were significantly and progressively associated with a higher risk for the primary outcome compared to the reference category. This finding was independent of the presence of T2D (overall *p*-interaction = 0.23; Fig. 1).

Supplementary Figure S3 shows all the independent predictors for the primary outcome in the overall population.

#### Secondary outcomes

Supplementary Table S6 report IR and IRR for each outcome.

**Fig. 2:**
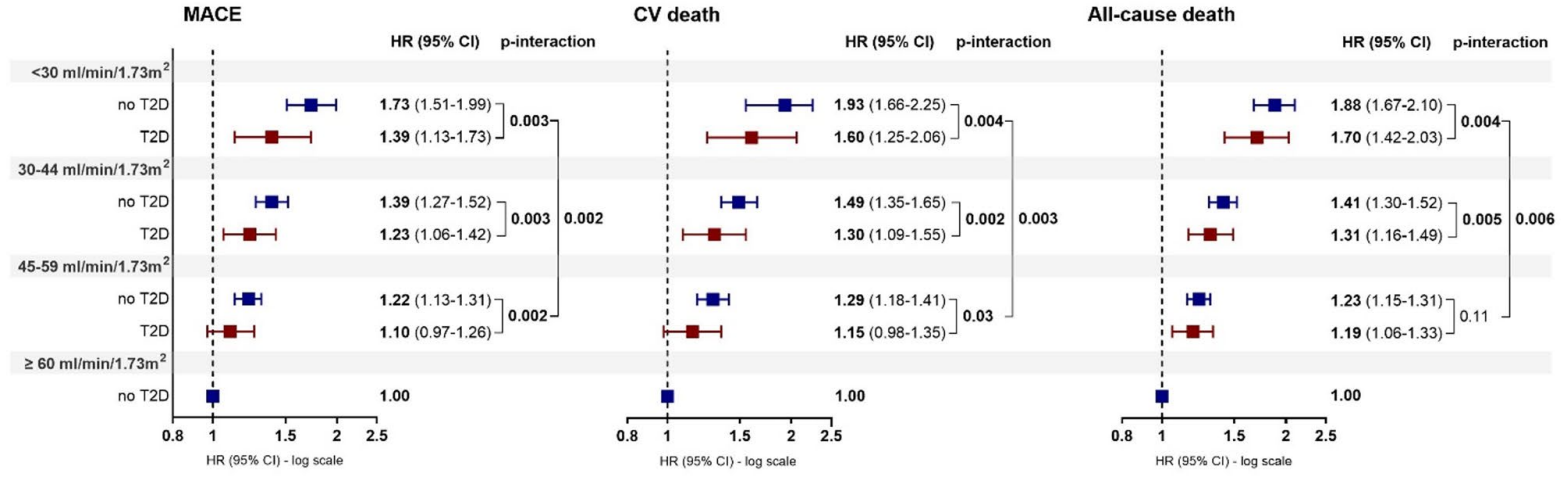
Association of eGFR ranges with MACE, CV death, all-cause death in the overall population according to T2D status. HR of each eGFR range (< 30, 30–44, 45–59 ml/min/1.73 m^2^) is relative to the eGFR reference category ≥ 60 ml/min/1.73 m^2^ of non T2D group (HR = 1). HHF, hospitalization for heart failure; CV, cardiovascular; eGFR, estimated glomerular filtration rate; T2D, Type 2 Diabetes; HR, Hazard Ratio; CI, confidence interval

The eGFR-related risk of MACE was progressively higher for lower eGFR categories, with significant higher hazards in the group without T2D compared to those with T2D (*p*-interaction < 0.01; Fig. 2, panel A). Similar findings were observed regarding CV death (Fig. 2*, panel B).* Lower eGFR ranges were also associated to an increased risk of all-cause death, with eGFR < 30 and 30–44 ml/min/1.73 m^2^ showing significant lower hazards in T2D patients (*p*-interaction < 0.01; Fig. 2, panel C).

No associations were found between eGFR ranges and stroke/TIA in both groups (Supplementary table S7). Lower eGFR, except for eGFR 45–59 ml/min/1.73m^2^, were significantly associated with a higher risk for MI, with no significant interaction between T2D and eGFR (Supplementary table S8).

Repeated HHF occurred more often in T2D group, with an IRR of 1.60 (95%CI 1.49–1.71, *p* < 0.001). There was a significant association of eGFR 30–44 and 45–59 ml/min/1.73 m^2^ with repeated HHF independently of T2D status (Supplementary table S9).

### Subgroup analysis

Supplementary Table S10 shows absolute numbers of events, event rates, and IR of outcomes in T2D vs non-T2D group across eGFR in each EF subgroup.

#### Primary outcome

**Fig. 3:**
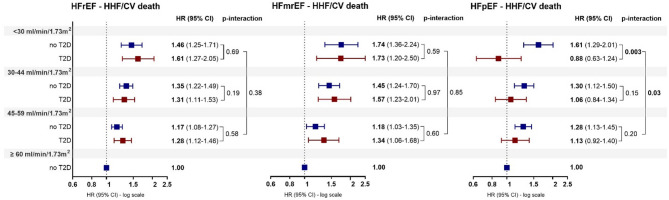
Primary outcome—subgroup analysis. Association of eGFR ranges with HHF/CV death in HFrEF, HFmrEF and HFpEF according to T2D. HR of each eGFR range (< 30, 30–44, 45–59 ml/min/1.73m^2^) is relative to the eGFR reference category ≥ 60 ml/min/1.73m^2^ of non T2D group (HR = 1). HHF, heart failure hospitalization; CV, cardiovascular; eGFR, estimated glomerular filtration rate; T2D, Type 2 Diabetes; HR, Hazard Ratio; CI, confidence interval

Consistent with what we found in the overall population, when analyzing the primary outcome by EF, we found a significant association with lower eGFR ranges in HFrEF and HFmrEF, regardless of T2D status. Conversely, in HFpEF, lower eGFR ranges were significantly associated with a progressively higher risk of the primary outcome in non-T2D patients, whereas no association was found in T2D group and eGFR < 30 ml/min/1.73m^2^ showed a significantly lower hazard in T2D patients compared to non-T2D (*p*-interaction < 0.01; Fig. 3).

#### MACE, CV death and all-cause death

When stratified by EF, lower eGFR ranges were progressively associated with a higher risk for MACE, CV death and all-cause death in each EF subgroup. A significant interaction between eGFR and TD2 with respect to these outcomes was confirmed in HFrEF, with eGFR 30–44 ml/min/1.73 m^2^ and 45–59 ml/min/1.73m^2^ showing significantly lower hazards in T2D compared to non-T2D patients (Supplementary Table S11). In HFmrEF and HFpEF subgroups, no significant interaction was found between T2D and eGFR for each of these outcomes (Supplementary tables S12–S13).

## Discussion

The main finding from our study was that decreased kidney function in HF was associated with a progressively higher risk of HHF/CV death, as expected, and T2D was not a risk modifier of this association.

Although patients with T2D had a higher overall event rate, the association between lower kidney function categories and adverse outcomes was consistent across diabetes status, indicating that diabetes did not modify the impact of renal impairment on prognosis in our population.

CKD was also associated with a higher risk of MACE, CV death and all-cause mortality; however, interestingly, for these outcomes the risk was higher in patients without T2D compared to those with.

These findings should be interpreted with caution due to overlapping confidence intervals and the potential influence of survivor or treatment biases. Patients with T2D often undergo more frequent clinical monitoring and earlier initiation of therapies with established cardiorenal benefits, such as SGLT2-i and GLP1-RA, which may mitigate the excess risk traditionally associated with diabetes. In contrast, individuals without T2D may undergo less systematic screening for renal dysfunction and have lower exposure to these cardiorenal-protective therapies.

Previous literature aligns with the role of CKD as a predictor of clinical outcomes in HF. Of particular relevance is a meta-analysis by Fox Cs et al. from 2012, which examined the associations between CKD and mortality across 30 general population and high-CV risk cohorts, and 13 CKD cohorts, encompassing a total of 1,024,977 individuals. This analysis revealed, in line with our results, that relative mortality risks by eGFR remained largely consistent regardless of the presence or absence of diabetes [15]. However, it is worth noting that the aforementioned study cohort had a lower proportion of patients with T2D compared to ours (13% vs 22%) and no subgroup analysis was conducted across EF. In our study, over one-third of the population had mildly to severely decreased kidney function, and the distribution of CKD stages was in line with earlier reports [16]. Additionally, CKD was more common in T2D patients than non-T2D, as already known [17, 18]. The role of CKD as an independent predictor of adverse outcomes in HF can be summarized in two key aspects, i.e. the shared pathophysiology of HF and CKD and the lower implementation of HF therapies in CKD, and both are probably on top of the existence of T2D [19]. Our findings also align with those recently reported by Jensen et al. in a large Norwegian registry study, which showed that CKD diagnosis was associated with substantial morbidity and mortality, both in patients with and without T2D [20].

Some additional insights can be offered by our EF-stratified analysis. The proportion of patients with HFpEF was slightly higher compared to previous registry-based studies,^22^ which may reflect its increasing prevalence due to better diagnosis and clinicians awareness [21, 22]. Additionally, HFpEF subgroup had a higher proportion of CKD compared to HFrEF and HFmrEF. It has been previously shown that HFpEF is associated with higher prevalence of CKD [23], which is thought to be driven by hemodynamic, neurohormonal and inflammatory pathways [24]. Within HFpEF subgroup, CKD was even more common in T2D patients, supporting the notion that comorbidities play a pivotal role especially in HFpEF [25, 26]. Previous studies have proposed that the impact of CKD in terms of severity and outcomes may vary in HFpEF [23, 27]. CKD may develop in association with HFpEF, due to shared underlying causes [28], whereas in HFrEF and HFmrEF it may be a consequence of the HF syndrome itself and its severity, developing secondary to hemodynamic perturbation of HF [23]. In conclusion, whether T2D may affect CKD-related outcomes in HFpEF remains to be clarified.

In our study, the association between eGFR ranges and MACE, CV death and all-cause mortality was stronger in non-T2D than T2D patients, albeit still statistically significant. Consistent with this finding, Fox et al. found that lower eGFR was associated with CV and all-cause mortality in individuals without diabetes or hypertension [15]. Accordingly, a prospective long-term cohort study, conducted as part of the Tehran Lipid and Glucose Study, revealed that non-diabetic patients experienced a higher average decline in eGFR compared to those with T2D [29]. This could be counterintuitive, since it is well-established that diabetes is a strong risk factor for CKD. However, it is worth noting several aspects. First, non-diabetic kidney disease accounts for almost three quarters of all cases of CKD [30]. Moreover, patients with diabetes might have more medical encounters and therefore are more strictly monitored, and glucose-lowering drugs recently adopted as first line therapy in high-CV risk T2D patients, such as SGLT2i and glucagon-like peptide-1 receptor agonists (GLP-1 RA), have demonstrated a favorable impact on renal outcomes [31–33]. In HF, SGLT2i are now standard of care and GLP-1 RA have shown some benefits [34–37]. Recently, the non-steroidal mineralocorticoid receptor agonist finerenone, which had already been shown to reduce the risk of cardiorenal outcomes in patients with T2D, demonstrated a benefit on HF-related outcomes in patients with HFmrEF and HFpEF [11, 38]. Finally, receiving glucose-lowering treatment possibly has a nefroprotective effect which is not granted in patients without diabetes. Overall, our findings underscore the importance of systematic CKD screening and implementation of cardiorenal-protective therapies in HF patients, regardless of diabetic status.

## Strengths and limitations

To our knowledge, this is the first large, long-term, cohort study to assess the effect modification of T2D on cardiovascular outcomes and mortality in an unselected and contemporary HF population according to CKD categorization and in HF subgroups. Our population was reasonably well treated as regards HF, possibly limiting the confounding effect of medications.

Some limitations should be acknowledged. Due to the observational design of our study, despite extensive adjustment, residual confounding is unavoidable, and no causal inference can be made. Some of the subgroups were numerically small, thus our results should be interpreted with caution. We defined CKD according to eGFR, even though the current definition also includes measurement of albuminuria and two consecutive eGFR estimates [39]. However, data on albuminuria in non-T2D patients were unavailable. We acknowledge that the lack of detailed information on the underlying causes of CKD may have restricted the evaluation of the differential prognostic impact of specific CKD subtypes. Our cohort may not be completely representative of the entire Swedish population with HF and our results might not be extensively generalizable or other geographical settings.

## Conclusions

In a contemporary HF cohort, decreased kidney function was associated with a progressively higher risk of HHF/CV death and T2D was not a risk modifier. In HFpEF, CKD showed a potential limited prognostic value when coexisting with T2D. Notably, our data suggested a potentially higher mortality risk among non-T2D possibly attributed to a lower use of cardio-renal protective therapies. These findings underscore the critical need for implementing renal protection in HF, irrespective of the presence of T2D. In clinical practice, this could be achieved by including screening for CKD in the structured HF follow-up. Further investigation is warranted to validate our observations and elucidate the underlying mechanisms and clinical implications.

## Supplementary Information

Below is the link to the electronic supplementary material.

Supplementary Material 1

## Data Availability

The datasets used and/or analysed during the current study are available from the corresponding author on reasonable request.

## Abbreviations

CKD: Chronic kidney disease
CV: Cardiovascular
HF: Heart failure
T2D: Type 2 diabetes
EF: Ejection fraction
HFrEF: HF with reduced EF
HFmrEF: HF with mildy-reduced EF
HFpEF: HF with preserved EF
eGFR: Estimated glomerular filtration rate
HHF: HF hospitalization
MACE: Major adverse CV event
SGLT2i: Sodium-glucose cotransporter-2 inhibitors
MRA: Non-steroidal mineralocorticoid receptor antagonists
SwedeHF: Swedish heart failure registry
NDR: National diabetes registry
NPR: National patient registry
ICD-10: International classification of diseases
MI: Non-fatal myocardial infarction
TIA: Transient ischemic attack
IR: Incidence rates
IRR: IR ratios
RAASi: Renin-angiotensin system inhibitors
ARNi: Angiotensin receptor-neprilysin inhibitors
