# Supplementary material for: Impact of type 2 diabetes on the relationship between chronic kidney disease and cardiovascular outcomes in heart failure across ejection fraction: observational study from the Swedish heart failure and the Swedish National diabetes registries
Source: Cardiovasc Diabetol. 2025 Dec 1;24:452. doi: 10.1186/s12933-025-02998-w (PMC12670795; doi:10.1186/s12933-025-02998-w)
Supplement: Supplementary file 1 — Supplementary Material 1 [file 12933_2025_2998_MOESM1_ESM.docx]

**Figure S1**. Flow chart reporting cohort selection.

75,843 records in SwedeHF from

1 January 2017 to 31 December 2021

Excluded:

● N = 19,640 EF missing

● N = 595 FU ≤ 14 days *

● N = 13,995 multiple registratoins

● N = 773 type 1, 3 or other/unknown types of diabetes

● N = 2,937 T2D and no registration in NDR +/- 6 months from index date

● N = 1,255 eGFR missing

● N = 8 patients who took insulin for unspecified reasons without T2D diagnosis

● N = 43 patients who took other hypoglycemic drugs for unspecified reasons without T2D diagnosis

**36,597** unique patients meeting the enrollment criteria

8,053 patients with T2D

28,544 patients without T2D

*In order to avoid immortal time bias, patients without at least 14 days follow-up were excluded, because of use of medications being defined as prescription until up to 14 days after the index date.

**Abbreviations**: EF: Ejection Fraction, FU: follow-up, T2D: type 2 diabetes, NDR: National Diabetes Registry, eGFR: estimated glomerular filtration rate

**Figure S2**. Bar graph of eGFR classes distribution according to T2D status.

**
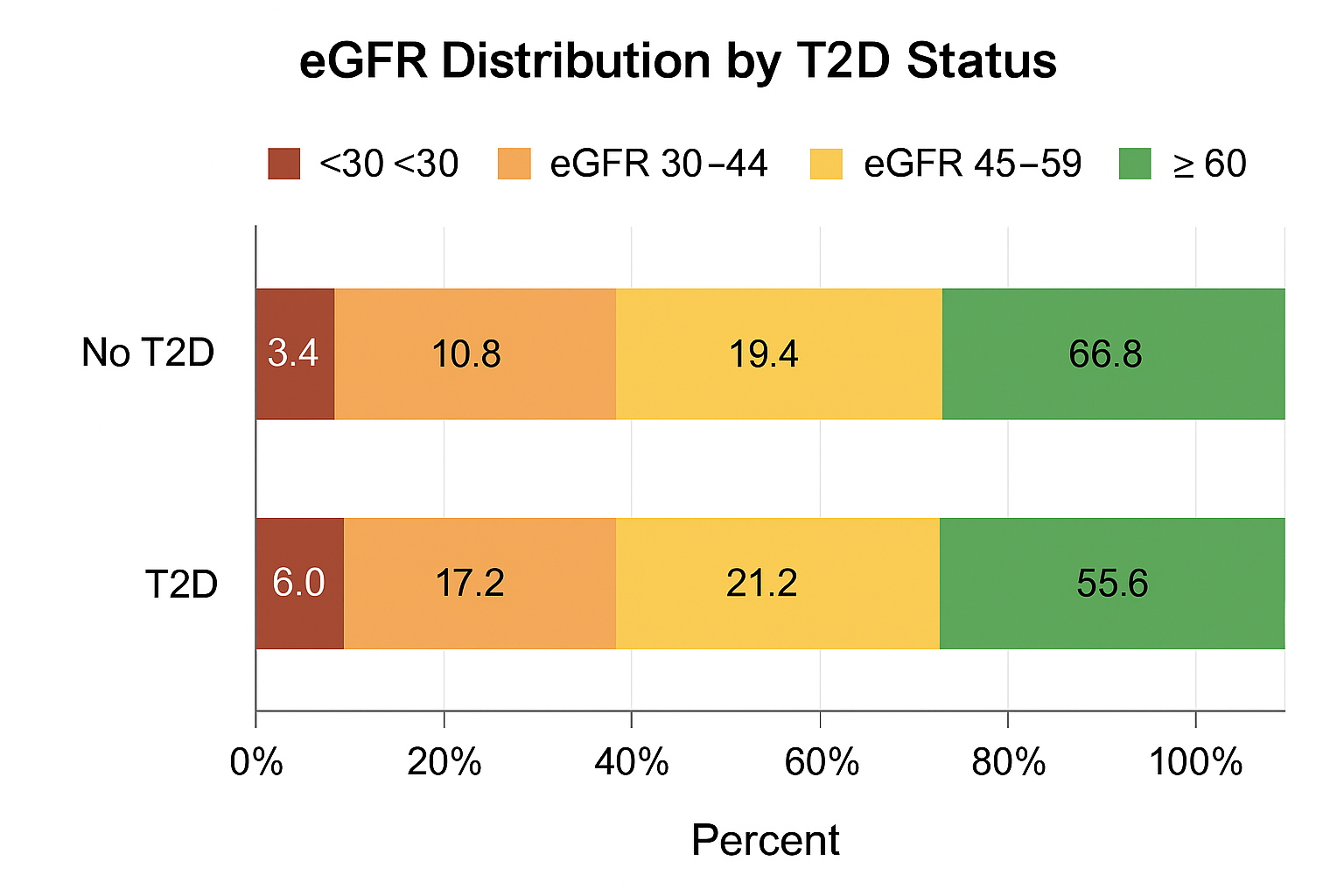
**

**Abbreviations:** eGFR, estimated glomerular filtration rate; T2D, type 2 diabetes

**Figure S3**. Predictors of the primary outcome in the overall population.

**
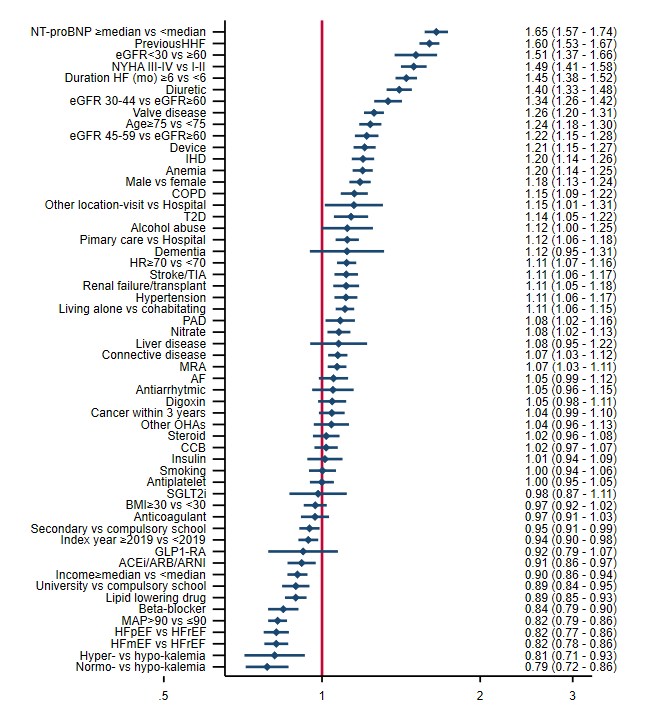
**

**HR (95% CI)**

**higher risk HHF/CV death**

**Abbreviations:** HHF, heart failure hospitalization; CV, cardiovascular; eGFR, estimated glomerular filtration rate; NYHA, New York Heart Association; HFrEF, heart failure with reduced ejection fraction; HFmrEF, heart failure with mildly reduced ejection fraction; HFpEF, heart failure with preserved ejection fraction; NT-proBNP, N-terminal pro B-type natriuretic peptide; MAP, mean arterial pressure; BMI, body mass index; COPD, chronic obstructive pulmonary disease; ACE-I, Angiotensin Converting Enzyme Inhibitors; ARB, Angiotensin Receptor Blockers; ARNI, angiotensin receptor-neprilysin inhibitors; MRA, mineralocorticoid receptor antagonists; SGLT2i, sodium-glucose cotransporter 2 inhibitors; GLP-1 RA, glucagon-like peptide 1 receptor agonists; HR, hazard ratio; CI, confidence interval.

**Table S1**. Variable definitions.

| **Variables** | **Definition** |
| --- | --- |
| EF | SwedeHF |
| Gender | SwedeHF |
| Age | SwedeHF |
| Smoke | SwedeHF |
| HF duration | SwedeHF |
| NYHA class | SwedeHF |
| MAP | SwedeHF |
| Heart rate | SwedeHF |
| Anemia | SwedeHF at index, haemoglobin <120 g/l in females and <130 g/l in males |
| Potassium | SwedeHF |
| eGFR | SwedeHF |
| NT-proBNP | SwedeHF |
| BMI | SwedeHF |
| Follow-up in nurse-led clinic | SwedeHF |
| Family type | Statistics Sweden |
| Education | Statistics Sweden |
| Income | Statistics Sweden |
| AF | SwedeHF at index (history of atrial fibrillation or ECG showing atrial fibrillation) or NPR in any position (ICD-10 code: I48) |
| COPD | Diagnosis in NPR in any position (ICD-10 codes: J40-4) |
| Arterial hypertension | Diagnosis in SwedeHF at index or in NPR in any position (ICD-10 codes: I10-I15) |
| PAD | Diagnosis in NPR in any position (ICD-10 codes: I70-I73) |
| IHD | Diagnosis in SwedeHF at index or in NPR in any position (ICD-10 codes: 410-4, I20-I25; procedure codes: FNA, FNB, FNC, FND, FNE, FNF, FNH, Z951, Z955). |
| Stroke/TIA | Diagnosis in NPR in any position (ICD-10 codes: 430-4, 438, I60-4, I690-4, G45) |
| Renal failure / dialysis / renal transplant | Diagnosis in the NPR in any position (ICD-10 codes: N17-9, Z491, Z492 OP: KAS00, KAS10, KAS20, DR014, DR015, DR016, DR020, DR012, DR013, DR023, DR024, TJA33, TJA35) |
| Muscoloskeletal disease within 3 years | Diagnosis in the NPR in any position within 3 years (ICD-10 codes:M) |
| Liver disease | Diagnosis in NPR in any position (ICD-10 codes: B18, I85, I864, I982, K70, K710, K711, K713-7, K72-4, K760, K762-9) |
| Dementia | Diagnosis in the NPR in any position within 3 years (ICD-10 codes:F00-4, R54) |
| History of cancer within 3 years | Diagnosis in the NPR in any position (ICD-10 codes: C) |
| Alcohol abuse | Diagnosis in the NPR in any position (ICD-10 codes:E244, E52, F10, G312, G621, G721, I426, K292, K70, K860, O354, P043, Q860, T51, Z502, Z714 Ekod:Y90, Y91) |
| Valve disease | Diagnosis in SwedeHF at index or in NPR in any position (ICD-10 codes: ICD: I05-8, I34-9, Q22, Q230-3, Q230-3, Q235-9, Z952-4) |
| Devices | SwedeHF |
| All medications | PDR; prescribed during a period of 5 months before index date and 14 days after index date |
| **Outcomes** |  |
| Hospitalization for HF | From NPR as main diagnosis (ICD-10 codes:I110, I130, I132, I255, I420, I423, I425, I426, I427, I428, I429, I43, I50, J81, K761, R570, 414W, 425E, 425F, 425G, 425H, 425W, 425X, 428) |
| Hospitalization for stroke/TIA | From NPR as main diagnosis (ICD-10 codes: I60-4, G45) |
| Hospitalization for myocardial infarction | From NPR as main diagnosis (ICD-10 codes: I21,122) |
| Cardiovascular death | From Cause of Death Registry: ICD-10 codes: I, J81, K761, R570, G45 |
| All-cause mortality | From Cause of Death Registry, any ICD-10 code |

**Abbreviations**: EF, ejection fraction; SwedeHF, Swedish Heart Failure Registry; NYHA, New York Heart Association; HF, heart failure; MAP, mean arterial pressure; eGFR, estimated glomerular filtration rate; NT-proBNP, N-terminal pro B-type natriuretic peptide; AF, atrial fibrillation; BMI, body mass index; COPD, chronic obstructive pulmonary disease; PAD, Peripheral artery disease; IHD, ischemic heart disease; TIA, transient ischemic attack; NPR: National Patient Register; PDR, Prescribed Drug Register.

**Table S2**. Percentage of missing data.

| **Variables** | **Missing (%)** |
| --- | --- |
| **Demographics** | |
| Age | 0 |
| Sex | 0 |
| Index year | 0 |
| Follow-up location | 2.9 |
| **Clinical variables** | |
| eGFR | 0 |
| EF | 0 |
| NYHA class | 22.8 |
| BMI | 21.4 |
| MAP | 2.2 |
| Heart Rate | 2.9 |
| NT-proBNP | 17.4 |
| Potassium | 0.7 |
| **Medical history (%)** | |
| Type 2 diabetes | 0 |
| Smoke | 26.5 |
| Alcohol abuse | 0 |
| HF duration | 3.3 |
| Previous HHF | 0 |
| Acute renal failure/dialysis/renal transplant | 0 |
| Atrial fibrillation | 0 |
| Hypertension | 0 |
| Peripheral artery disease | 0 |
| Ischemic heart disease | 0 |
| Valvular disease | 0 |
| Cerebrovascular disease | 0 |
| COPD | 0 |
| Cancer within 3 years | 0 |
| Liver disease | 0 |
| Musculoskeletal disease | 0 |
| Dementia | 0 |
| Anemia | 9.5 |
| Devices | 0.5 |
| **Medication use** | |
| Beta blockers | 0 |
| MRA | 0 |
| RASi or ARNi | 0 |
| Calcium-chanel blockers | 0 |
| Digoxin | 0 |
| Antiarrhythmics | 0 |
| Diuretics | 0 |
| Nitrates | 0 |
| Lipid-lowering drugs | 0 |
| Anticoagulants | 0 |
| Anti-platelets | 0.29 |
| Steroids | 0 |
| Insulin | 0 |
| SGLT2-i | 0 |
| GLP1-RA | 0 |
| Other Oral Hypoglycemic drugs | 0 |
| **Social economic characteristics** | |
| Family setting | 0.1 |
| Educational level | 1.3 |
| Income | 0.1 |

**Abbreviations**: eGFR, estimated glomerular filtration rate; EF, ejection fraction; NYHA, New York Heart Association; BMI, body mass index; MAP, mean arterial pressure; NT-proBNP, N-terminal pro-B-type natriuretic peptide; HF, heart failure; HHF, HF hospitalization; COPD, chronic obstructive pulmonary disease; MRA, mineralocorticoid receptor antagonist; RASi, renin angiotensin converting enzyme inhibitors; ARNI, angiotensin receptor-neprilysin inhibitor; SGLT2-I, sodium glucose cotransporter 2; GLP1-RA: glucagon like peptide 1 receptor agonist.

**Table S3**. Additional baseline characteristics not displayed in Table 1.

|  | **Overall** | | | | **eGFR <30**  **ml/min/1.73 m2** | | | **eGFR 30-44 ml/min/1.73 m2** | | | **eGFR 45-59 ml/min/1.73 m2** | | | **eGFR ≥60**  **ml/min/1.73 m2** | | |
| --- | --- | --- | --- | --- | --- | --- | --- | --- | --- | --- | --- | --- | --- | --- | --- | --- |
|  | **Overall** | **no T2D** | **T2D** | **p-value** | **no T2D** | **T2D** | **p-value** | **no T2D** | **T2D** | **p-value** | **no T2D** | **T2D** | **p-value** | **no T2D** | **T2D** | **p-value** |
| Index year, n (%)^b^ |  |  |  | <0.001 |  |  | 0.25 |  |  | 0.99 |  |  | 0.89 |  |  | 0.005 |
| 2017-2018 | 16613 (45.4) | 12811 (44.9) | 3802 (47.2) |  | 488 (50.9) | 258 (54.1) |  | 1556 (50.3) | 696 (50.3) |  | 2600 (48.1) | 826 (48.2) |  | 8167 (42.8) | 2022 (45.1) |  |
| 2019-2021 | 19984 (54.6) | 15733 (55.1) | 4251 (52.8) |  | 471 (49.1) | 219 (45.9) |  | 1537 (49.7) | 688 (49.7) |  | 2810 (51.9) | 886 (51.8) |  | 10915 (57.2) | 2458 (54.9) |  |
| Location of visit, n (%)^a,b^ |  |  |  | 0.001 |  |  | 0.11 |  |  | 0.11 |  |  | 0.34 |  |  | <0.001 |
| Hospital | 26725 (75.2) | 20970 (75.7) | 5755 (73.7) |  | 575 (63.0) | 310 (68.6) |  | 1930 (64.9) | 896 (67.6) |  | 3647 (69.9) | 1187 (71.7) |  | 14818 (79.6) | 3362 (76.8) |  |
| Primary care | 7846 (22.1) | 6002 (21.7) | 1844 (23.6) |  | 309 (33.8) | 128 (28.3) |  | 959 (32.2) | 385 (29.1) |  | 1421 (27.2) | 426 (25.7) |  | 3313 (17.8) | 905 (20.7) |  |
| Other | 956 (2.7) | 746 (2.7) | 210 (2.7) |  | 29(3.2) | 14(3.1) |  | 87(2.9) | 44(3.3) |  | 153 (2.9) | 43(2.6) |  | 477 (2.6) | 109 (2.5) |  |
| **Clinical variables** | | | | | | | | | | | | | | | | |
| Potassium (mmol/L), n(%)^a,b^ |  |  |  | <0.001 |  |  | 0.79 |  |  | 0.011 |  |  | <0.001 |  |  | <0.001 |
| Hypokalemia (<3.5 mmol/L) | 1203 (3.3) | 934 (3.3) | 269 (3.4) |  | 37(3.9) | 15(3.2) |  | 140 (4.6) | 41(3.0) |  | 195 (3.6) | 71(4.2) |  | 562 (3.0) | 142 (3.2) |  |
| Normokalemia (3.5-5 mmol/L) | 33904 (93.3) | 26582 (93.8) | 7322 (91.7) |  | 804 (84.3) | 404 (84.9) |  | 2728 (88.9) | 1222 (89.0) |  | 4952 (92.2) | 1522 (89.5) |  | 18098 (95.5) | 4174 (94.0) |  |
| Hyperkalemia (>5 mmol/L) | 1224 (3.4) | 827 (2.9) | 397 (5.0) |  | 113 (11.8) | 57 (12.0) |  | 199 (6.5) | 110 (8.0) |  | 222 (4.1) | 107 (6.3) |  | 293 (1.5) | 123 (2.8) |  |
| Heart rate >70 bpm, n (%)^a,b^ | 17810 (50.1) | 13590 (49.0) | 4220 (53.9) | <0.001 | 458 (49.2) | 227 (48.9) | 0.91 | 1531 (50.9) | 667 (49.6) | 0.42 | 2641 (50.2) | 852 (51.1) | 0.52 | 8960 (48.4) | 2474 (56.8) | <0.001 |
| MAP >90 mmHg, n (%)^a,b^ | 18295 (51.1) | 14345 (51.4) | 3950 (50.1) | 0.040 | 398 (43.1) | 222 (47.8) | 0.095 | 1344 (44.3) | 615 (45.1) | 0.60 | 2531 (47.8) | 777 (46.2) | 0.25 | 10072 (54.0) | 2336 (53.3) | 0.46 |
| **Medical History** | | | | | | | | | | | | | | | | |
| Valvular disease, n (%)^b^ | 8801 (24.0) | 6963 (24.4) | 1838 (22.8) | 0.004 | 330 (34.4) | 124 (26.0) | 0.001 | 1022 (33.0) | 386 (27.9) | <0.001 | 1591 (29.4) | 406 (23.7) | <0.001 | 4020 (21.1) | 922 (20.6) | 0.47 |
| Anemia, n (%)^ab^ | 10326 (31.2) | 7447 (28.8) | 2879 (39.5) | <0.001 | 587 (65.0) | 306 (68.3) | 0.23 | 1265 (44.9) | 627 (50.0) | 0.003 | 1686 (34.1) | 658 (42.3) | <0.001 | 3909 (22.8) | 1288 (31.9) | <0.001 |
| Liver disease, n (%)^b^ | 839 (2.3) | 613 (2.1) | 226 (2.8) | <0.001 | 23(2.4) | 19(4.0) | 0.093 | 60(1.9) | 40(2.9) | 0.047 | 73 (1.3) | 37 (2.2) | 0.018 | 457 (2.4) | 130 (2.9) | 0.050 |
| Cancer within 3 years, n (%)^b^ | 4795 (13.1) | 3744 (13.1) | 1051 (13.1) | 0.88 | 173 (18.0) | 84 (17.6) | 0.84 | 512 (16.6) | 221 (16.0) | 0.62 | 803 (14.8) | 239 (14.0) | 0.37 | 2256 (11.8) | 507 (11.3) | 0.34 |
| Connective disease within 3 years, n (%)^b^ | 11615 (31.7) | 8789 (30.8) | 2826 (35.1) | <0.001 | 410 (42.8) | 195 (40.9) | 0.50 | 1139 (36.8) | 553 (40.0) | 0.046 | 1825 (33.7) | 606 (35.4) | 0.21 | 5415 (28.4) | 1472 (32.9) | <0.001 |
| Dementia, n (%)^b^ | 374 (1.0) | 288 (1.0) | 86 (1.1) | 0.64 | 12(1.3) | 6(1.3) | 0.99 | 59(1.9) | 15(1.1) | 0.046 | 72(1.3) | 22(1.3) | 0.88 | 145 (0.8) | 43(1.0) | 0.18 |
| Devices (%)^a,b^ | 6813 (18.7) | 5113 (18.0) | 1700 (21.2) | <0.001 | 203 (21.5) | 102 (21.6) | 0.95 | 747 (24.4) | 336 (24.6) | 0.87 | 1169 (21.7) | 405 (23.8) | 0.082 | 2994 (15.8) | 857 (19.2) | <0.001 |
| **Concomitant medications** | | | | | | | | | | | | | | | | |
| Digoxin, n (%)^b^ | 4022 (11.0) | 3140 (11.0) | 882 (11.0) | 0.90 | 71(7.4) | 23(4.8) | 0.062 | 322 (10.4) | 127 (9.2) | 0.20 | 694 (12.8) | 187 (10.9) | 0.037 | 2053 (10.8) | 545 (12.2) | 0.007 |
| Antiarrhythmics, n (%)^b^ | 1718 (4.7) | 1388 (4.9) | 330 (4.1) | 0.004 | 67(7.0) | 19(4.0) | 0.024 | 221 (7.1) | 68(4.9) | 0.005 | 302 (5.6) | 84 (4.9) | 0.28 | 798 (4.2) | 159 (3.5) | 0.053 |
| Calcium-chanel blockers, n (%)^b^ | 7486 (20.5) | 4988 (17.5) | 2498 (31.0) | <0.001 | 314 (32.7) | 252 (52.8) | <0.001 | 675 (21.8) | 511 (36.9) | <0.001 | 1074 (19.9) | 536 (31.3) | <0.001 | 2925 (15.3) | 1199 (26.8) | <0.001 |
| Nitrates, n (%)^b^ | 7637 (20.9) | 5358 (18.8) | 2279 (28.3) | <0.001 | 240 (25.0) | 175 (36.7) | <0.001 | 679 (22.0) | 440 (31.8) | <0.001 | 1095 (20.2) | 498 (29.1) | <0.001 | 3344 (17.5) | 1166 (26.0) | <0.001 |
| Corticosteroids, n (%)^b^ | 4503 (12.3) | 3470 (12.2) | 1033 (12.8) | 0.11 | 202 (21.1) | 76 (15.9) | 0.020 | 453 (14.6) | 221 (16.0) | 0.25 | 718 (13.3) | 247 (14.4) | 0.22 | 2097 (11.0) | 489 (10.9) | 0.89 |
| Other Oral Hypoglicemic Drugs, n (%)^b^ | 5080 (13.9) | 0(0.0) | 5080 (63.1) | <0.001 | 0(0.0) | 163 (34.2) | <0.001 | 0(0.0) | 654 (47.3) | <0.001 | 0(0.0) | 1041 (60.8) | <0.001 | 0(0.0) | 3222 (71.9) | <0.001 |

**Table S4**. Distribution of eGFR categories in the overall population and according to T2D status.

|  | **eGFR<30** | **eGFR 30-44** | **eGFR 45-59** | **eGFR ≥60** | Total |
| --- | --- | --- | --- | --- | --- |
| **Overall HF, n (%)** | 1436 (4.0%) | 4477 (12.2%) | 7122 (19.4%) | 23562 (64.4%) | 36597 (100%) |
| No T2D, n (%) | 959 (3.4%) | 3093 (10.8%) | 5410 (19.0%) | 19082 (66.8%) | 28544 (100%) |
| T2D, n (%) | 477 (6.0%) | 1384 (17.2%) | 1712 (21.2%) | 4480 (55.6%) | 8053 (100%) |

**Abbreviations**: eGFR: estimated glomerular filtration rate; HF, heart failure; T2D, type 2 diabetes.

**Table S5**. Distribution of eGFR categories across ejection fraction and according to T2D status.

|  | **eGFR<30** | **eGFR 30-44** | **eGFR 45-59** | **eGFR ≥60** | Total |
| --- | --- | --- | --- | --- | --- |
| **HFrEF, n (%)** | 709 (3.7%) | 2081 (10.9%) | 3534 (18.4%) | 12851 (67.0%) | 19175 (100%) |
| No T2D, n (%) | 499 (3.3%) | 1443 (9.6%) | 2663 (17.8%) | 10370 (69.3%) | 14975 (100%) |
| T2D, n (%) | 210 (5%) | 638 (15.2%) | 871 (20.7%) | 2481 (59.1%) | 4200 (100%) |
| **HFmrEF, n (%)** | 325 (3.5%) | 1085 (11.6%) | 1784 (19.2%) | 6110 (65.7%) | 9304 (100%) |
| No T2D, n (%) | 204 (2.8%) | 749 (10.1%) | 1375 (18.6%) | 5060 (68.5%) | 7388 (100%) |
| T2D, n (%) | 121 (6.3%) | 336 (17.5%) | 409 (21.4%) | 1050 (54.8%) | 1916 (100%) |
| **HFpEF, n (%)** | 402 (5.0%) | 1311 (16.1%) | 1804 (22.2%) | 4601 (56.7%) | 8118 (100%) |
| No T2D, n (%) | 256 (4.1%) | 901 (14.6%) | 1372 (22.2%) | 3652 (59.1%) | 6181 (100%) |
| T2D, n (%) | 146 (7.5%) | 410 (21.2%) | 432 (22.3%) | 949 (49%) | 1937 (100%) |

**Abbreviations**: eGFR: estimated glomerular filtration rate; HFrEF: heart failure with reduced ejection fraction; HFmrEF: heart failure with mildly reduced ejection fraction; HFpEF: heart failure with preserved ejection fraction.

**Table S6**. Incidence rates of outcomes in T2D and non-T2D groups across eGFR ranges in the overall population.

| **Outcome** | **Number of events per 100 person-years** | | **IRR** | **95% Confidence Interval** | **p-value** |
| --- | --- | --- | --- | --- | --- |
|  | **No T2D** | **T2D** |  |  |  |
| **Primary composite** | **12.82** | **19.36** | **1.51** | **1.45-1.58** | **<0.001** |
| eGFR<30 | 38.09 | 41.74 | 1.10 | 0.94-1.28 | 0.24 |
| eGFR 30-44 | 26.65 | 30.94 | 1.16 | 1.06-1.27 | <0.01 |
| eGFR 45-59 | 17.41 | 24.64 | 1.41 | 1.29-1.53 | <0.001 |
| eGFR ≥ 60 | 9.11 | 13.46 | 1.48 | 1.38-1.58 | <0.001 |
| M-H |  |  | 1.35 | 1.29-1.40 | <0.001 |
| **HF hospitalization** | **9.99** | **16.02** | **1.60** | **1.53-1.68** | **<0.001** |
| eGFR <30 | 27.32 | 32.57 | 1.19 | 0.99-1.42 | 0.05 |
| eGFR 30-44 | 20.63 | 25.77 | 1.25 | 1.12-1.39 | <0.001 |
| eGFR 45-59 | 13.27 | 20.78 | 1.57 | 1.42-1.72 | <0.001 |
| eGFR ≥ 60 | 7.27 | 11.06 | 1.52 | 1.412-1.64 | <0.001 |
| M-H |  |  | 1.44 | 1.37-1.51 | <0.001 |
| **CV death** | **5.07** | **6.29** | **1.24** | **1.16-1.33** | **<0.001** |
| eGFR <30 | 18.77 | 15.20 | 0.81 | 0.65-0.99 | <0.05 |
| eGFR 30-44 | 11.97 | 10.05 | 0.84 | 0.73-0.96 | <0.05 |
| eGFR 45-59 | 7.37 | 7.36 | 0.99 | 0.87-1.14 | 0.99 |
| eGFR ≥ 60 | 3.01 | 4.19 | 1.39 | 1.25-1.55 | <0.001 |
| M-H |  |  | 1.05 | 0.98-1.13 | <0.001 |
| **Myocardial infarction** | **0.89** | **2.05** | **2.31** | **2.02-2.63** | **<0.001** |
| eGFR <30 | 2.31 | 4.83 | 2.09 | 1.30-3.37 | <0.01 |
| eGFR 30-44 | 3.26 | 3.26 | 2.29 | 1.69-3.11 | <0.001 |
| eGFR 45-59 | 1.03 | 2.01 | 1.95 | 1.45-2.62 | <0.001 |
| eGFR ≥ 60 | 0.72 | 1.52 | 2.10 | 1.72-2.55 | <0.001 |
| M-H |  |  | 2.11 | 1.84-2.41 | 0.90 |
| **Stroke/TIA** | **1.57** | **2.25** | **1.43** | **1.27-1.61** | **<0.001** |
| eGFR <30 | 2.46 | 3.25 | 1.32 | 0.78-2.21 | 0.27 |
| eGFR 30-44 | 2.37 | 2.66 | 1.12 | 0.84-1.50 | 0.41 |
| eGFR 45-59 | 1.91 | 2.61 | 1.36 | 1.07-1.74 | <0.05 |
| eGFR ≥ 60 | 1.34 | 1.93 | 1.44 | 1.22-1.69 | <0.001 |
| M-H |  |  | 1.35 | 1.20-1.52 | 0.52 |
| **MACE** | **7.01** | **9.73** | **1.39** | **1.32-1.47** | **<0.001** |
| eGFR <30 | 21.73 | 21.12 | 0.97 | 0.80-1.17 | 0.76 |
| eGFR 30-44 | 14.70 | 14.81 | 1.01 | 0.89-1.13 | 0.90 |
| eGFR 45-59 | 9.68 | 11.00 | 1.14 | 1.01-1.27 | 0.03 |
| eGFR ≥ 60 | 4.72 | 7.04 | 1.49 | 1.37-1.62 | <0.001 |
| M-H |  |  | 1.22 | 1.15-1.29 | <0.001 |
| **All-cause death** | **9.14** | **12.25** | **1.34** | **1.28-1.41** | **<0.001** |
| eGFR <30 | 32.49 | 31.23 | 0.96 | 0.83-1.12 | 0.60 |
| eGFR 30-44 | 19.75 | 19.22 | 0.97 | 0.88-1.08 | 0.59 |
| eGFR 45-59 | 12.57 | 14.04 | 1.12 | 1.01-1.23 | <0.05 |
| eGFR ≥ 60 | 5.91 | 8.21 | 1.39 | 1.29-1.5 | <0.001 |
|  |  |  | 1.15 | 1.10-1.21 | <0.001 |
| **Recurrent HHF** | **N.A.** | **N.A.** | **1.60** | **1.49-1.71** | **<0.001** |
| eGFR <30 |  |  | 1.14 | 0.93-1.38 | 0.20 |
| eGFR 30-44 |  |  | 1.22 | 1.08-1.38 | <0.01 |
| eGFR45-49 |  |  | 1.47 | 1.31-1.65 | <0.001 |
| eGFR ≥ 60 |  |  | 1.56 | 1.42-1.72 | <0.001 |

**Abbreviations**: T2D, type 2 diabetes; IRR, incident rate ratio, eGFR, estimated glomerular filtration rate; HF, heart failure; CV, cardiovascular; TIA, transient ischemic attack; MACE, major cardiovascular events; HHF, hospitalization for heart failure.

**Table S7**. Association of eGFR ranges with stroke/TIA in the overall population according to T2D.

|  | **Hazard Ratio** | **95% Confidential Interval** | **p-value** | **p-interaction** |
| --- | --- | --- | --- | --- |
| **eGFR <30** | | | | |
| Overall | 1.10 | 0.82-1.47 | 0.53 |  |
| No T2D | 1.13 | 0.79-1.64 | 0.50 | 0.91 |
| T2D | 1.04 | 0.64-1.70 | 0.86 |  |
| **eGFR 30-44** | | | | |
| Overall | 1.08 | 0.91-1.28 | 0.37 |  |
| No T2D | 1.12 | 0.91-1.37 | 0.29 | 0.49 |
| T2D | 0.98 | 0.71-1.33 | 0.88 |  |
| **eGFR 45-59** | | | | |
| Overall | 1.05 | 0.92-1.21 | 0.45 |  |
| No T2D | 1.03 | 0.88-1.22 | 0.69 | 0.80 |
| T2D | 1.08 | 0.84-1.40 | 0.54 |  |

**Abbreviations**: eGFR, estimated glomerular filtration rate; T2D, type 2 diabetes.

**Table S8**. Association of eGFR ranges with myocardial infarction in the overall population according to T2D.

|  | **Hazard Ratio** | **95% Confidential Interval** | **p-value** | **p-interaction** |
| --- | --- | --- | --- | --- |
| **eGFR <30** | | | | |
| **Overall** | **1.57** | **1.16-2.13** | **0.004** |  |
| No T2D | 1.64 | 1.08-2.48 | 0.02 | 0.52 |
| T2D | 1.39 | 0.88-2.20 | 0.16 |  |
| **eGFR 30-44** | | | | |
| **Overall** | **1.31** | **1.07-1.61** | **0.01** |  |
| No T2D | 1.28 | 0.97-1.69 | 0.08 | 0.99 |
| T2D | 1.34 | 0.98-1.84 | 0.07 |  |
| **eGFR 45-59** | | | | |
| Overall | 1.05 | 0.88-1.26 | 0.56 |  |
| No T2D | 1.07 | 0.86-1.34 | 0.55 | 0.66 |
| T2D | 1.02 | 0.76-1.37 | 0.90 |  |

**Abbreviations**: eGFR, estimated glomerular filtration rate; T2D, type 2 diabetes.

**Table S9**. Association of eGFR with repeated HHF in the overall HF population according to T2D.

|  | **Hazard Ratio** | **95% Confidential Interval** | **p-value** | **p-interaction** |
| --- | --- | --- | --- | --- |
| **eGFR <30** | | | | |
| Overall | 1.02 | 0.90-1.16 | 0.72 |  |
| No T2D | 1.04 | 0.88-1.22 | 0.64 | 0.31 |
| T2D | 1.02 | 0.82-1.26 | 0.89 |  |
| **eGFR 30-44** | | | | |
| **Overall** | **1.27** | **1.17-1.37** | **<0.001** |  |
| No T2D | 1.28 | 1.16-1.41 | <0.001 | 0.30 |
| T2D | 1.24 | 1.08-1.42 | 0.003 |  |
| **eGFR 45-59** | | | | |
| **Overall** | **1.15** | **1.08-1.23** | **<0.001** |  |
| No T2D | 1.13 | 1.05-1.22 | 0.002 | 0.61 |
| T2D | 1.22 | 1.08-1.37 | 0.001 |  |

**Abbreviations**: eGFR, estimated glomerular filtration rate; T2D, type 2 diabetes.

**Table S10**. Incidence rates of outcomes in T2D vs non-T2D group across eGFR in HFpEF, HFmrEF, HFrEF.

Obs: For the recurrent HF hospitalization the numbers of events are not given, since in binomial regression counts are the expected ones only.

| **Outcome** | **Absolute number of events** | | **Number of events per 100 person-years** | | **IRR** | **95% Confidence Interval** | **p-value** |
| --- | --- | --- | --- | --- | --- | --- | --- |
|  | **No T2D** | **T2D** | **No T2D** | **T2D** |  |  |  |
| **Primary composite outcome** | | | | | | | |
| Primary composite, HFrEF | 4165 | 1563 | 14.31 | 21.03 | 1.47 | 1.38-1.56 | <0.001 |
| eGFR <30 | 257 | 118 | 40.89 | 50.39 | 1.23 | 0.98-1.54 | 0.06 |
| eGFR 30-44 | 683 | 321 | 29.87 | 34.04 | 1.14 | 0.99-1.30 | 0.05 |
| eGFR 45-59 | 957 | 414 | 19.14 | 27.52 | 1.44 | 1.28-1.62 | <0.001 |
| eGFR ≥ 60 | 2268 | 710 | 10.70 | 14.94 | 1.39 | 1.28-1.52 | <0.001 |
| M-H |  |  |  |  | 1.34 | 1.26-1.42 | <0.05 |
| Primary composite, HFmrEF | 1557 | 619 | 9.84 | 16.80 | 1.71 | 1.55-1.87 | <0.001 |
| eGFR <30 | 100 | 67 | 33.13 | 43.11 | 1.30 | 0.94-1.79 | 0.09 |
| eGFR 30-44 | 315 | 156 | 24.01 | 29.84 | 1.24 | 1.02-1.51 | <0.05 |
| eGFR 45-59 | 400 | 146 | 14.14 | 20.13 | 1.42 | 1.17-1.72 | <0.001 |
| eGFR ≥ 60 | 742 | 250 | 6.52 | 10.96 | 1.68 | 1.45-1.94 | <0.001 |
| M-H |  |  |  |  | 1.46 | 1.33- 1.60 | 0.07 |
| Primary composite, HFpEF | 1645 | 684 | 13.12 | 18.55 | 1.41 | 1.29-1.55 | <0.001 |
| eGFR <30 | 131 | 70 | 37.34 | 31.62 | 0.85 | 0.62-1.14 | 0.26 |
| eGFR 30-44 | 361 | 181 | 24.05 | 27.38 | 1.14 | 0.95-1.36 | 0.16 |
| eGFR 45-59 | 461 | 170 | 17.67 | 22.53 | 1.27 | 1.06-1.52 | <0.05 |
| eGFR ≥ 60 | 692 | 263 | 8.56 | 12.83 | 1.50 | 1.29-1.73 | <0.001 |
| M-H |  |  |  |  | 1.26 | 1.15-1.38 | <0.01 |
| **HF hospitalization** | | | | | | | |
| HF hospitalization, HFrEF | 3359 | 1346 | 11.54 | 18.11 | 1.57 | 1.47-1.67 | <0.001 |
| eGFR <30 | 192 | 92 | 30.54 | 39.29 | 1.29 | 0.99-1.65 | 0.05 |
| eGFR 30-44 | 540 | 274 | 23.62 | 29.06 | 1.23 | 1.06.1.42 | <0.05 |
| eGFR 45-59 | 743 | 373 | 14.86 | 24.79 | 1.67 | 1.47-1.89 | <0.001 |
| eGFR ≥ 60 | 1884 | 607 | 8.90 | 12.77 | 1.43 | 1.31-1.57 | <0.001 |
| M-H |  |  |  |  | 1.44 | 1.35-1.53 | <0.01 |
| HF hospitalization, HFmrEF | 1159 | 491 | 7.32 | 13.32 | 1.82 | 1.63-2.02 | <0.001 |
| eGFR <30 | 64 | 56 | 21.20 | 36.03 | 1.70 | 1.16-2.47 | <0.01 |
| eGFR 30-44 | 240 | 131 | 18.29 | 25.06 | 1.37 | 1.10-1.70 | <0.01 |
| eGFR 45-59 | 298 | 112 | 10.53 | 15.44 | 1.47 | 1.17-1.83 | <0.01 |
| eGFR ≥ 60 | 557 | 192 | 4.89 | 8.41 | 1.72 | 1.45-2.03 | <0.001 |
| M-H |  |  |  |  | 1.56 | 1.40-1.73 | 0.34 |
| HF hospitalization, HFpEF | 1223 | 535 | 9.75 | 14.51 | 1.49 | 1.34-1.65 | <0.001 |
| eGFR <30 | 94 | 51 | 26.79 | 23.04 | 0.86 | 0.60-1.22 | 0.39 |
| eGFR 30-44 | 272 | 143 | 18.12 | 21.63 | 1.19 | 0.97-1.46 | 0.09 |
| eGFR 45-59 | 344 | 135 | 13.18 | 17.89 | 1.36 | 1.10-1.66 | <0.01 |
| eGFR ≥ 60 | 513 | 206 | 6.35 | 10.05 | 1.58 | 1.34-1.86 | <0.001 |
| M-H |  |  |  |  | 1.33 | 1.20-1.47 | <0.05 |
| **CV death** | | | | | | | |
| CV death, HFrEF | 1725 | 600 | 5.03 | 6.35 | 1.26 | 1.15-1.38 | <0.001 |
| eGFR <30 | 158 | 61 | 19.02 | 17.46 | 0.92 | 0.67-1.24 | 0.58 |
| eGFR 30-44 | 367 | 140 | 12.45 | 10.54 | 0.85 | 0.69-1.03 | 0.09 |
| eGFR 45-59 | 467 | 155 | 7.68 | 7.58 | 0.99 | 0.82-1.18 | 0.88 |
| eGFR ≥ 60 | 733 | 244 | 3.00 | 4.26 | 1.42 | 1.22- 1.64 | <0.001 |
| M-H |  |  |  |  | 1.09 | 0.99-1.19 | <0.001 |
| CV death, HFmrEF | 750 | 262 | 4.36 | 6.05 | 1.39 | 1.20-1.60 | <0.001 |
| eGFR <30 | 63 | 31 | 17.36 | 14.31 | 0.82 | 0.52-1.29 | 0.38 |
| eGFR 30-44 | 185 | 72 | 11.91 | 10.67 | 0.89 | 0.67-1.18 | 0.43 |
| eGFR 45-59 | 200 | 61 | 6.37 | 6.97 | 1.09 | 0.81-1.46 | 0.53 |
| eGFR ≥ 60 | 302 | 98 | 2.48 | 3.82 | 1.54 | 1.21-1.94 | <0.001 |
| M-H |  |  |  |  | 1.13 | 0.98-1.30 | <0.05 |
| CV death, HFpEF | 843 | 284 | 6.07 | 6.41 | 1.05 | 0.92-1.21 | 0.43 |
| eGFR <30 | 84 | 37 | 19.47 | 13.10 | 0.67 | 0.44-1.00 | <0.5 |
| eGFR 30-44 | 200 | 75 | 11.23 | 8.80 | 0.78 | 0.59-1.03 | 0.07 |
| eGFR 45-59 | 232 | 69 | 7.78 | 7.24 | 0.93 | 0.70-1.22 | 0.61 |
| eGFR ≥ 60 | 327 | 103 | 3.76 | 4.39 | 1.17 | 0.93-1.46 | 0.17 |
| M-H |  |  |  |  | 0.92 | 0.81-1.05 | <0.05 |
| **Myocardial infarction** | | | | | | | |
| Myocardial infarction, HFrEF | 313 | 169 | 0.92 | 1.83 | 1.98 | 1.63-2.39 | <0.001 |
| eGFR <30 | 20 | 10 | 2.46 | 2.93 | 1.19 | 0.50-2.66 | 0.64 |
| eGFR 30-44 | 48 | 42 | 1.66 | 3.28 | 1.98 | 1.27-3.05 | <0.01 |
| eGFR 45-59 | 67 | 34 | 1.12 | 1.69 | 1.52 | 0.97-2.33 | 0.05 |
| eGFR ≥ 60 | 178 | 83 | 0.73 | 1.48 | 2.01 | 1.53-2.62 | <0.001 |
| Myocardial infarction, HFmrEF | 134 | 103 | 0.79 | 2.45 | 3.12 | 2.39-4.07 | <0.001 |
| eGFR <30 | 9 | 14 | 2.51 | 6.80 | 2.71 | 1.09-7.10 | <0.05 |
| eGFR 30-44 | 7 | 23 | 0.45 | 3.54 | 7.83 | 3.25-21.62 | <0.001 |
| eGFR 45-59 | 32 | 23 | 1.04 | 2.73 | 2.63 | 1.47-4.65 | <0.01 |
| eGFR ≥ 60 | 86 | 43 | 0.71 | 1.72 | 2.41 | 1.63-3.52 | <0.001 |
| Myocardial infarction, HFpEF | 126 | 91 | 0.92 | 2.11 | 2.30 | 1.73-3.03 | <0.001 |
| eGFR <30 | 8 | 15 | 1.86 | 5.78 | 3.10 | 1.23-8.46 | <0.05 |
| eGFR 30-44 | 33 | 25 | 1.89 | 3.01 | 1.59 | 0.91-2.76 | 0.08 |
| eGFR 45-59 | 25 | 19 | 0.85 | 2.05 | 2.41 | 1.26-4.57 | <0.01 |
| eGFR ≥ 60 | 60 | 32 | 0.69 | 1.39 | 1.99 | 1.26-3.12 | <0.01 |
| **Stroke/TIA** | | | | | | | |
| Stroke/TIA, HFrEF | 481 | 198 | 1.43 | 2.15 | 1.50 | 1.27-1.78 | <0.001 |
| eGFR <30 | 18 | 11 | 2.21 | 3.21 | 1.45 | 0.62-3.25 | 0.33 |
| eGFR 30-44 | 70 | 36 | 2.44 | 2.79 | 1.14 | 0.74-1.73 | 0.52 |
| eGFR 45-59 | 113 | 50 | 1.91 | 2.54 | 1.33 | 0.93-1.87 | 0.10 |
| eGFR ≥ 60 | 280 | 101 | 1.17 | 1.81 | 1.55 | 1.22-1.95 | <0.001 |
| Stroke/TIA, HFmrEF | 263 | 96 | 1.56 | 2.28 | 1.46 | 1.43-1.85 | <0.01 |
| eGFR <30 | 8 | 9 | 2.30 | 4.30 | 1.87 | 0.64-5.58 | 0.21 |
| eGFR 30-44 | 34 | 15 | 2.24 | 2.25 | 1.01 | 0.51-1.90 | 0.97 |
| eGFR 45-59 | 49 | 24 | 1.58 | 2.82 | 1.77 | 1.04-2.95 | <0.05 |
| eGFR ≥ 60 | 172 | 48 | 1.44 | 1.93 | 1.33 | 0.95-1.85 | 0.08 |
| Stroke/TIA, HFpEF | 265 | 105 | 1.95 | 2.44 | 1.25 | 0.99-1.57 | 0.06 |
| eGFR <30 | 13 | 7 | 3.11 | 2.52 | 0.81 | 0.27-2.18 | 0.67 |
| eGFR 30-44 | 41 | 23 | 2.35 | 2.79 | 1.19 | 0.68-2.02 | 0.51 |
| eGFR 45-59 | 66 | 24 | 2.28 | 2.59 | 1.13 | 0.68-1.84 | 0.58 |
| eGFR ≥ 60 | 145 | 51 | 1.70 | 2.24 | 1.32 | 0.94-1.82 | 0.10 |
| **MACE** | | | | | | | |
| MACE, HFrEF | 2280 | 863 | 6.86 | 9.59 | 1.40 | 1.29-1.51 | <0.001 |
| eGFR <30 | 174 | 69 | 21.81 | 20.58 | 0.94 | 0.70-1.25 | 0.69 |
| eGFR 30-44 | 444 | 193 | 15.80 | 15.47 | 0.98 | 0.82-1.16 | 0.81 |
| eGFR 45-59 | 582 | 216 | 9.95 | 11.19 | 1.12 | 0.96-1.32 | 0.14 |
| eGFR ≥ 60 | 1080 | 385 | 4.55 | 7.02 | 1.54 | 1.37-1.73 | <0.001 |
| M-H |  |  |  |  | 1.23 | 1.14-1.33 | <0.001 |
| MACE, HFmrEF | 1046 | 393 | 6.26 | 9.60 | 1.53 | 1.36-1.72 | <0.001 |
| eGFR <30 | 72 | 45 | 20.89 | 22.67 | 1.08 | 0.73-1.60 | 0.66 |
| eGFR 30-44 | 202 | 97 | 13.35 | 15.12 | 1.13 | 0.88-1.45 | 0.32 |
| eGFR 45-59 | 260 | 87 | 8.56 | 10.61 | 1.24 | 0.96-1.59 | 0.09 |
| eGFR ≥ 60 | 512 | 164 | 4.34 | 6.74 | 1.55 | 1.30-1.86 | <0.001 |
| M-H |  |  |  |  | 1.31 | 1.17-1.48 | 0.10 |
| MACE, HFpEF | 1115 | 427 | 8.30 | 10.17 | 1.22 | 1.09-1.37 | <0.001 |
| eGFR <30 | 93 | 53 | 22.29 | 20.62 | 0.92 | 0.65-1.31 | 0.65 |
| eGFR 30-44 | 241 | 109 | 14.09 | 13.55 | 0.96 | 0.76-1.21 | 0.74 |
| eGFR 45-59 | 296 | 99 | 10.34 | 10.95 | 1.06 | 0.83-1.33 | 0.62 |
| eGFR ≥ 60 | 485 | 166 | 5.74 | 7.43 | 1.29 | 1.08-1.54 | <0.01 |
| M-H |  |  |  |  | 1.09 | 0.98-1.23 | 0.13 |
| **All-cause death** | | | | | | | |
| All-cause death, HFrEF | 2875 | 1076 | 8.39 | 11.39 | 1.36 | 1.26-1.46 | <0.001 |
| eGFR <30 | 269 | 109 | 32.38 | 31.20 | 0.96 | 0.76-1.21 | 0.75 |
| eGFR 30-44 | 558 | 239 | 18.92 | 17.99 | 0.95 | 0.81-1.11 | 0.51 |
| eGFR 45-59 | 727 | 277 | 11.96 | 13.54 | 1.13 | 0.98-1.30 | 0.08 |
| eGFR ≥ 60 | 1321 | 451 | 5.41 | 7.87 | 1.46 | 1.31-1.62 | <0.001 |
| M-H |  |  |  |  | 1.18 | 1.10-1.27 | <0.001 |
| All-cause death, HFmrEF | 1441 | 509 | 8.37 | 11.76 | 1.40 | 1.27-1.55 | <0.001 |
| eGFR <30 | 106 | 70 | 29.20 | 32.31 | 1.11 | 0.81-1.51 | 0.51 |
| eGFR 30-44 | 306 | 135 | 19.70 | 20.00 | 1.01 | 0.82-1.25 | 0.88 |
| eGFR 45-59 | 385 | 114 | 12.27 | 13.03 | 1.06 | 0.85-1.31 | 0.57 |
| eGFR ≥ 60 | 644 | 190 | 5.29 | 7.42 | 1.40 | 1.18-1.65 | <0.01 |
| M-H |  |  |  |  | 1.17 | 1.06-1.29 | 0.05 |
| All-cause death, HFpEF | 1661 | 646 | 11.96 | 14.57 | 1.22 | 1.11-1.33 | <0.001 |
| eGFR <30 | 153 | 86 | 35.47 | 30.45 | 0.86 | 0.65-1.12 | 0.26 |
| eGFR 30-44 | 377 | 175 | 21.17 | 20.53 | 0.97 | 0.80-1.16 | 0.74 |
| eGFR 45-59 | 421 | 153 | 14.12 | 16.06 | 1.14 | 0.94-1.37 | 0.17 |
| eGFR ≥ 60 | 710 | 232 | 8.17 | 9.90 | 1.21 | 1.04-1.41 | <0.05 |
| M-H |  |  |  |  | 1.08 | 0.98-1.18 | 0.07 |
| **Recurrent HF Hospitalization** | | | | | | | |
| Recurrent HF hospitalization, HFrEF | - | - | - | - | 1.54 | 1.42-1.68 | <0.001 |
| eGFR <30 | - | - | - | - | 1.05 | 0.78-1.40 | 0.75 |
| eGFR 30-44 | - | - | - | - | 1.12 | 0.94-1.33 | 0.21 |
| eGFR45-49 | - | - | - | - | 1.57 | 1.34-1.83 | <0.001 |
| eGFR ≥ 60 | - | - | - | - | 1.55 | 1.38-1.75 | <0.001 |
| Recurrent HF hospitalization, HFmrEF | - | - | - | - | 1.76 | 1.54-2.01 | <0.001 |
| eGFR <30 | - | - | - | - | 1.63 | 1.11-2.38 | <0.05 |
| eGFR 30-44 | - | - | - | - | 1.41 | 1.10-1.80 | <0.01 |
| eGFR45-49 | - | - | - | - | 1.27 | 0.98-1.64 | 0.07 |
| eGFR ≥ 60 | - | - | - | - | 1.66 | 1.33-2.06 | <0.001 |
| Recurrent HF hospitalization, HFpEF | - | - | - | - | 1.51 | 1.33-1.71 | <0.001 |
| eGFR <30 | - | - | - | - | 1.13 | 0.79-1.63 | 0.50 |
| eGFR 30-44 | - | - | - | - | 1.31 | 1.04-1.65 | <0.05 |
| eGFR45-49 | - | - | - | - | 1.33 | 1.06-1.68 | <0.05 |
| eGFR ≥ 60 | - | - | - | - | 1.52 | 1.23-1.87 | <0.001 |

**Abbreviations**: eGFR, estimated glomerular filtration rate; T2D, type 2 diabetes; IRR, incident rate ratio; HF, heart failure; HFrEF, heart failure with reduced ejection fraction; HFmrEF, heart failure with mildly reduced ejection fraction; HFpEF, heart failure with preserved ejection fraction; M-H, Mantel–Haenszel; CV, cardiovascular; TIA, transient ischemic attack; MACE, major cardiovascular events.

**Table S11**. Association of eGFR with MACE, CV death and all-cause death in HFrEF.

|  | **Hazard Ratio** | **95% Confidential Interval** | **p-value** | **p-interaction** |
| --- | --- | --- | --- | --- |
| **MACE** | | | | |
| **eGFR <30** | | | | |
| **Overall** | **1.63** | **1.38-1.92** | **<0.001** |  |
| No T2D | 1.69 | 1.39-2.05 | <0.001 | 0.06 |
| T2D | 1.38 | 1.00-1.88 | 0.047 |  |
| **eGFR 30-44** | | | | |
| **Overall** | **1.39** | **1.25-1.55** | **<0.001** |  |
| No T2D | 1.47 | 1.29-1.67 | <0.001 | **0.005** |
| T2D | 1.19 | 0.97-1.46 | 0.10 |  |
| **eGFR 45-59** | | | | |
| **Overall** | **1.23** | **1.12-1.35** | **<0.001** |  |
| No T2D | 1.28 | 1.15-1.42 | <0.001 | **0.02** |
| T2D | 1.07 | 0.89-1.28 | 0.47 |  |
| **CV death** | | | | |
| **eGFR <30** | | | | |
| **Overall** | **1.84** | **1.54-2.21** | **<0.001** |  |
| No T2D | 1.85 | 1.49-2.29 | <0.001 | 0.24 |
| T2D | 1.71 | 1.20-2.43 | 0.003 |  |
| **eGFR 30-44** | | | | |
| **Overall** | **1.40** | **1.23-1.58** | **<0.001** |  |
| No T2D | 1.48 | 1.28-1.72 | <0.001 | **0.01** |
| T2D | 1.19 | 0.93-1.53 | 0.17 |  |
| **eGFR 45-59** | | | | |
| **Overall** | **1.29** | **1.16-1.43** | **<0.001** |  |
| No T2D | 1.35 | 1.19-1.53 | <0.001 | **0.02** |
| T2D | 1.09 | 0.87-1.35 | 0.45 |  |
| **All-cause death** | | | | |
| **eGFR <30** | | | | |
| **Overall** | **1.84** | **1.61-2.12** | **<0.001** |  |
| No T2D | 1.89 | 1.60-2.23 | <0.001 | 0.07 |
| T2D | 1.68 | 1.29-2.19 | <0.001 |  |
| **eGFR 30-44** | | | | |
| **Overall** | **1.31** | **1.19-1.45** | **<0.001** |  |
| No T2D | 1.38 | 1.23-1.55 | <0.001 | **0.005** |
| T2D | 1.17 | 0.97-1.42 | 1.10 |  |
| **eGFR 45-59** | | | | |
| **Overall** | **1.23** | **1.13-1.34** | **<0.001** |  |
| No T2D | 1.26 | 1.15-1.39 | <0.001 | **0.04** |
| T2D | 1.12 | 0.95-1.32 | 0.16 |  |

**Abbreviations**: eGFR, estimated glomerular filtration rate; T2D, type 2 diabetes; HFrEF, heart failure with reduced ejection fraction; MACE, major cardiovascular events; CV, cardiovascular.

**Table S12**. Association of eGFR with MACE, CV death and all-cause death in HFmrEF.

|  | **Hazard Ratio** | **95% Confidential Interval** | **p-value** | **p-interaction** |
| --- | --- | --- | --- | --- |
| **MACE** | | | | |
| **eGFR <30** | | | | |
| **Overall** | **1.74** | **1.36-2.23** | **<0.001** |  |
| No T2D | 1.90 | 1.41-2.57 | <0.001 | 0.26 |
| T2D | 1.47 | 0.93-2.34 | 0.10 |  |
| **eGFR 30-44** | | | | |
| **Overall** | **1.40** | **1.19-1.63** | **<0.001** |  |
| No T2D | 1.38 | 1.14-1.67 | 0.001 | 0.94 |
| T2D | 1.44 | 1.06-1.95 | 0.01 |  |
| **eGFR 45-59** | | | | |
| **Overall** | **1.19** | **1.04-1.37** | **0.01** |  |
| No T2D | 1.16 | 0.99-1.37 | 0.07 | 0.79 |
| T2D | 1.26 | 0.95-1.68 | 0.11 |  |
| **CV death** | | | | |
| **eGFR <30** | | | | |
| **Overall** | **2.05** | **1.55-2.72** | **<0.001** |  |
| No T2D | 2.24 | 1.61-3.11 | <0.001 | 0.15 |
| T2D | 1.69 | 0.96-2.99 | 0.07 |  |
| **eGFR 30-44** | | | | |
| **Overall** | **1.76** | **1.46-2.11** | **<0.001** |  |
| No T2D | 1.74 | 1.40-2.15 | <0.001 | 0.86 |
| T2D | 1.81 | 1.25-2.63 | 0.002 |  |
| **eGFR 45-59** | | | | |
| **Overall** | **1.33** | **1.12-1.57** | **0.001** |  |
| No T2D | 1.29 | 1.06-1.57 | 0.01 | 0.69 |
| T2D | 1.48 | 1.04-2.11 | 0.03 |  |
| **All-cause death** | | | | |
| **eGFR <30** | | | | |
| **Overall** | **1.93** | **1.57-2.38** | **<0.001** |  |
| No T2D | 1.98 | 1.55-2.54 | <0.001 | 0.58 |
| T2D | 2.04 | 1.37-3.03 | <0.001 |  |
| **eGFR 30-44** | | | | |
| **Overall** | **1.57** | **1.37-1.80** | **<0.001** |  |
| No T2D | 1.54 | 1.31-1.80 | <0.001 | 0.77 |
| T2D | 1.73 | 1.31-2.27 | <0.001 |  |
| **eGFR 45-59** | | | | |
| **Overall** | **1.30** | **1.156-1.47** | **<0.001** |  |
| No T2D | 1.27 | 1.11-1.46 | 0.001 | 0.60 |
| T2D | 1.45 | 1.12-1.87 | 0.005 |  |

**Abbreviations**: eGFR, estimated glomerular filtration rate; T2D, type 2 diabetes; HFmrEF, heart failure with mildly reduced ejection fraction; MACE, major cardiovascular events; CV, cardiovascular.

**Table S13**. Association of eGFR with MACE, CV death and all-cause death in HFpEF.

|  | **Hazard Ratio** | **95% Confidential Interval** | **p-value** | **p-interaction** |
| --- | --- | --- | --- | --- |
| **MACE** | | | | |
| **eGFR <30** | | | | |
| **Overall** | **1.66** | **1.33-2.06** | **<0.001** |  |
| No T2D | 1.72 | 1.32-2.23 | <0.001 | 0.12 |
| T2D | 1.49 | 0.99-2.23 | 0.05 |  |
| **eGFR 30-44** | | | | |
| **Overall** | **1.26** | **1.09-1.47** | **0.002** |  |
| No T2D | 1.25 | 1.05-1.50 | 0.01 | 0.17 |
| T2D | 1.14 | 0.85-1.52 | 0.38 |  |
| **eGFR 45-59** | | | | |
| **Overall** | **1.15** | **1.01-1.31** | **0.04** |  |
| No T2D | 1.15 | 0.98-1.34 | 0.08 | 0.23 |
| T2D | 1.08 | 0.83-1.41 | 0.57 |  |
| **CV death** | | | | |
| **eGFR <30** | | | | |
| **Overall** | **1.80** | **1.41-2.30** | **<0.001** |  |
| No T2D | 1.86 | 1.40-2.47 | <0.001 | 0.09 |
| T2D | 1.57 | 0.97-2.56 | 0.07 |  |
| **eGFR 30-44** | | | | |
| **Overall** | **1.32** | **1.11-1.58** | **0.002** |  |
| No T2D | 1.34 | 1.09-1.65 | 0.005 | 0.10 |
| T2D | 1.16 | 0.81-1.66 | 0.42 |  |
| **eGFR 45-59** | | | | |
| **Overall** | **1.20** | **1.03-1.40** | **0.02** |  |
| No T2D | 1.20 | 1.00-1.43 | **0.046** | 0.34 |
| T2D | 1.13 | 0.81-1.58 | 0.46 |  |
| **All-cause death** | | | | |
| **eGFR <30** | | | | |
| **Overall** | **1.78** | **1.50-2.13** | **<0.001** |  |
| No T2D | 1.84 | 1.49-2.27 | <0.001 | 0.10 |
| T2D | 1.71 | 1.24-2.37 | 0.001 |  |
| **eGFR 30-44** | | | | |
| **Overall** | **1.36** | **1.20-1.54** | **<0.001** |  |
| No T2D | 1.38 | 1.19-1.59 | <0.001 | 0.16 |
| T2D | 1.26 | 1.00-1.60 | 0.05 |  |
| **eGFR 45-59** | | | | |
| **Overall** | **1.15** | **1.03-1.29** | **0.01** |  |
| No T2D | 1.14 | 1.00-1.29 | 0.05 | 0.62 |
| T2D | 1.15 | 0.92-1.43 | 0.22 |  |

**Abbreviations**: eGFR, estimated glomerular filtration rate; T2D, type 2 diabetes; HFpEF, heart failure with preserved ejection fraction; MACE, major cardiovascular events; CV, cardiovascular.
